# Type IV Pili Are a Critical Virulence Factor in Clinical Isolates of Paenibacillus thiaminolyticus

**DOI:** 10.1128/mbio.02688-22

**Published:** 2022-11-14

**Authors:** Christine Hehnly, Aiqin Shi, Paddy Ssentongo, Lijun Zhang, Albert Isaacs, Sarah U. Morton, Nicholas Streck, Petra Erdmann-Gilmore, Igor Tolstoy, R. Reid Townsend, David D. Limbrick, Joseph N. Paulson, Jessica E. Ericson, Michael Y. Galperin, Steven J. Schiff, James R. Broach

**Affiliations:** a Institute for Personalized Medicine, Department of Biochemistry and Molecular Biology, The Pennsylvania State University College of Medicine, Hershey, Pennsylvania, USA; b Department of Public Health Sciences, The Pennsylvania State University College of Medicine, Hershey, Pennsylvania, USA; c Department of Medicine, Hershey Medical Center, Hershey, Pennsylvania, USA; d Department of Population and Quantitative Health Sciences, School of Medicine, Case Western Reserve University, Cleveland, Ohio, USA; e Division of Neurosurgery, Department of Clinical Neuroscience, University of Calgary, Calgary, Alberta, Canada; f Division of Newborn Medicine, Boston Children’s Hospital and Department of Pediatrics, Harvard Medical School, Boston, Massachusetts, USA; g Department of Pathology and Laboratory Medicine Division of Clinical Pathology, The Pennsylvania State University College of Medicine, Hershey, Pennsylvania, USA; h Department of Medicine, Washington University School of Medicine, St. Louis, Missouri, USA; i National Center for Biotechnology Information, National Library of Medicine, National Institutes of Health, Bethesda, Maryland, USA; j Department of Neurological Surgery, Washington University School of Medicine, St. Louis, Missouri, USA; k Division of Pediatric Infectious Disease, The Pennsylvania State University College of Medicine, Hershey, Pennsylvania, USA; l Department of Neurosurgery, Yale University, New Haven, Connecticut, USA; University of Illinois at Chicago

**Keywords:** *Paenibacillus thiaminolyticus*, postinfectious hydrocephalus, type IV pilus, virulence factors

## Abstract

Hydrocephalus, the leading indication for childhood neurosurgery worldwide, is particularly prevalent in low- and middle-income countries. Hydrocephalus preceded by an infection, or postinfectious hydrocephalus, accounts for up to 60% of hydrocephalus in these areas. Since many children with hydrocephalus suffer poor long-term outcomes despite surgical intervention, prevention of hydrocephalus remains paramount. Our previous studies implicated a novel bacterial pathogen, Paenibacillus thiaminolyticus, as a causal agent of neonatal sepsis and postinfectious hydrocephalus in Uganda. Here, we report the isolation of three *P. thiaminolyticus* strains, Mbale, Mbale2, and Mbale3, from patients with postinfectious hydrocephalus. We constructed complete genome assemblies of the clinical isolates as well as the nonpathogenic *P. thiaminolyticus* reference strain and performed comparative genomic and proteomic analyses to identify potential virulence factors. All three isolates carry a unique beta-lactamase gene, and two of the three isolates exhibit resistance in culture to the beta-lactam antibiotics penicillin and ampicillin. In addition, a cluster of genes carried on a mobile genetic element that encodes a putative type IV pilus operon is present in all three clinical isolates but absent in the reference strain. CRISPR-mediated deletion of the gene cluster substantially reduced the virulence of the Mbale strain in mice. Comparative proteogenomic analysis identified various additional potential virulence factors likely acquired on mobile genetic elements in the virulent strains. These results provide insight into the emergence of virulence in *P. thiaminolyticus* and suggest avenues for the diagnosis and treatment of this novel bacterial pathogen.

## INTRODUCTION

Hydrocephalus is one of the most common brain disorders in children globally and the most common indication for pediatric neurosurgery (1, 2). A serious infection such as neonatal sepsis often precedes hydrocephalus (3), and postinfectious hydrocephalus (PIH) accounts for up to 60% of the nearly 400,000 children who develop hydrocephalus each year, principally in low- and middle-income countries (1, 3, 4). PIH remains a leading cause of neurological morbidity and mortality worldwide despite recent clinical efforts to optimize treatment (5, 6).

Strategies to prevent PIH have been thwarted for two principal reasons. First, standard clinical evaluation often fails to identify the pathogen(s) responsible for the underlying infectious episodes that precede PIH (7), precluding targeted treatment of the underlying infections. Second, the pathophysiologic changes that lead to PIH infection remain unknown (8). Unfortunately, even children who undergo technically expert surgical treatment for hydrocephalus can suffer poor long-term outcomes (6, 9). Therefore, major advances in the health of these children will require preventing infection by targeting both the underlying pathogens and their routes of infection (10–12), as well as improving treatment of infections with more appropriate antibiotics and adjunctive therapies that can reduce the likelihood of subsequently developing hydrocephalus.

We recently identified and isolated a novel bacterial strain, Paenibacillus thiaminolyticus Mbale, that likely causes PIH in a significant fraction of cases in Uganda (13). We showed that *P. thiaminolyticus* Mbale is lethal to mice following peritoneal injection, whereas injection of the *P. thiaminolyticus* reference strain, NRRL B-4156^T^, does not cause lethality. *Paenibacillus* species have been isolated and studied from various sources, particularly in agricultural and industrial contexts (14). Although some species, such as Paenibacillus alvei and Paenibacillus larvae, are known to cause widespread disease in honeybees (15), until recently only anecdotal cases of human disease associated with *Paenibacillus* have been reported (16–21). We recently confirmed our initial report implicating *P. thiaminolyticus* as a causative agent of PIH (13) by identifying *P. thiaminolyticus* infection in the cerebrospinal fluid (CSF) of 41% of 205 infants with PIH (22). Finally, we isolated two additional clinical *P. thiaminolyticus* strains from patients with PIH, and their properties are described in this report.

Pathogenic bacteria typically encode specific proteins, or virulence factors, that aid in their ability to survive and propagate in their hosts. Given the differential virulence between the reference strain and the Mbale strain in mice, we surmised that the clinical isolates from our patients carry virulence factors that are absent in the nonpathogenic reference strain. We addressed this issue, as described in this report, by comparing the genome, transcriptome and proteome of our clinical isolates to those of the reference strain. Using a novel CRISPR-Cas9 gene deletion system, we confirmed that a type IV pilus (T4P) locus identified with comparative proteogenomics is a critical virulence factor in at least one of the clinical isolates. These results provide insight into the mechanism of virulence of *P. thiaminolyticus* and suggest avenues for diagnosis and treatment of this novel bacterial pathogen.

## RESULTS

### Complete assemblies of clinical isolates and reference strain.

**FIG 1:**
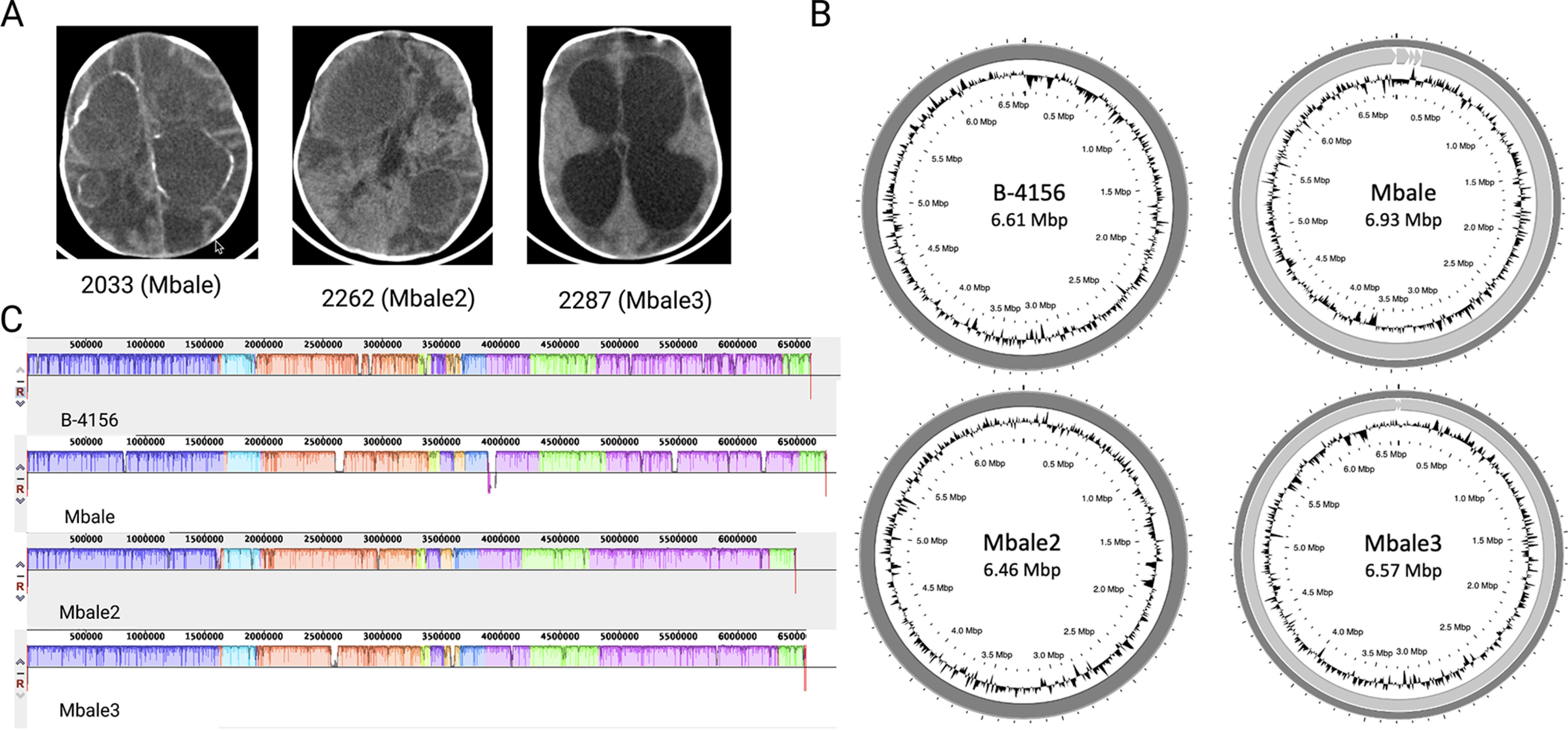
Genomic characterization of clinical isolates of *P. thiaminolyticus*. (A) Computerized tomography scans from the three infants with postinfectious hydrocephalus from whose CSF the three clinical isolates were recovered. The first two images, from Patient 2033 (clinical strain Mbale) and Patient 2262 (clinical strain Mbale2), were taken prior to surgery and demonstrate loculations and calcified abscess formation. The third image, from Patient 2287 (clinical strain Mbale3), was taken during surgery for hydrocephalus and also shows evidence of extensive brain parenchyma damage. (B) Complete assemblies of the reference strain of *P. thiaminolyticus*, B-4156^T^, and three clinical isolates, Mbale, Mbale2, and Mbale3, obtained using long- and short-read sequencing. The B-4156 and Mbale3 genomes each consists of one continuous contig, while the Mbale and Mbale2 genomes each consist of one large contig plus additional contigs (Table 1). (C) Alignment with MAUVE of the clinical isolates’ genomes to that of the B-4156 reference strain identified 12 locally colinear blocks, which are indicated by the different colors. White regions within the colored regions represent regions of low sequence similarity.

We previously described the isolation of a novel bacterial strain, which we designated Paenibacillus thiaminolyticus Mbale, from the CSF of a patient with PIH (13). This strain was identified as *P. thiaminolyticus* based on matrix-assisted laser desorption ionization–time-of-flight (MALDI-TOF) analysis, rRNA gene similarity, average nucleotide identity and phylogenetic analysis (13, 23). Here, we report two additional isolates, designated Mbale2 and Mbale3, that we recovered from the CSF of two additional patients with PIH and identified as *P. thiaminolyticus* by MALDI-TOF analysis. Computerized tomography scans of the brains of the infants from which these strains were recovered (Fig. 1A) document the extensive cerebral damage associated with infection by these isolates.

We previously provided the complete genome assemblies of the Mbale strain and the *P. thiaminolyticus* reference strain (23). The reference strain assembly yielded a complete 6,613-kbp chromosome, while the initial clinical Mbale strain required optical mapping to construct one complete chromosome from three contigs assembled by long-read sequencing; three shorter contigs remained unmapped (Fig. 1B; Table 1). PHASTER (24) analysis identified 2 of the 3 unmapped contigs as either complete or incomplete phage sequences (see Fig. S1B in the supplemental material). The third unmapped contig likely constitutes an insertion in the chromosome that is flanked by extended repeated sequences, rendering it unmappable by short and long-read sequencing, but localized by optical genome mapping.

**TABLE 1:** Summary of features *P. thiaminolyticus* strains, including those determined with PATRIC (29) and RefSeq (28)^*a*^

Feature	Result or value for:							
	B-4156		Mbale		Mbale2		Mbale3	
Assembly features								
BioProject accession no.	PRJNA552222		PRJNA552221		PRJNA799352		PRJNA799352	
No. of:								
Chromosomes	1		1		1		1	
Uncharacterized contigs	0		1		0		0	
Pred extrachromosomal phages	0		2		0		1	
Size (kbp)	6,613		6,932		6,460		6,573	
GC content (%)	53.6		53.3		53.7		53.5	
Genomic features (no.)	PATRIC	RefSeq	PATRIC	RefSeq	PATRIC	RefSeq	PATRIC	RefSeq
CDS	6,605	5,710	7,206	6,258	6,563	ND	7,015	ND
Repeat region	129	5	162	11	120	ND	116	ND
CRISPR repeat	124	0	153	0	31	ND	107	ND
CRISPR spacer	119	0	144	0	28	ND	99	ND
tRNA	83	83	86	86	16	ND	83	ND
Regulatory	83	30	31	31	0	ND	0	ND
Misc RNA	27	0	24	0	0	ND	0	ND
Misc binding			10	10	0	ND	0	ND
CRISPR array	24	0	9	0	3	ND	8	ND
rRNA	1	24	4	24	16	ND	15	ND
ncRNA	0	3	0	3	0	ND	0	ND
tmRNA	0	1	0	1	0	ND	0	ND
Protein features [no. (%)]								
Hypothetical proteins	2,661 (40)	810	3,171	1,138	2,750	ND	3,002	ND
Proteins with functional assignment	3,944 (60)	4,900	4,035	5,120	3,813	ND	4,013	ND

a CDS, coding sequences; CRISPR, clustered regularly interspaced short palindromic repeats; misc, miscellaneous; ncRNA, noncoding RNA; ND, not determined; pred, predicted; tmRNA transfer-messenger RNA. Annotations were obtained by RASTtk.

10.1128/mbio.02688-22.1 FIG S1 Phage regions identified in each clinical isolate. The results from PHASTER for strains (A) B-4156, (B) Mbale, (C) Mbale2, and (D) Mbale3. The colors correspond to the score that PHASTER assigns to the phage identification region, indicating an intact phage (green), questionable (blue), and incomplete (red). This score reflects the extent of presence of appropriate proteins for a functional phage. Download FIG S1, DOCX file, 0.3 MB. Copyright © 2022 Hehnly et al. 2022 Hehnly et al. https://creativecommons.org/licenses/by/4.0/ This content is distributed under the terms of the Creative Commons Attribution 4.0 International license.

Genome sequencing of the two new isolates revealed a single 6,460-kbp contig for the Mbale2 strain and two contigs, 6,561 kbp and 12 kbp, for the Mbale3 strain (Table 1; Fig. 1B). PHASTER analysis identified the 12-kbp contig of Mbale3 as an incomplete phage genome (Fig. S1; Table 1). We further confirmed the species assignment as *P. thiaminolyticus* by average nucleotide identity (ANI) (25). The average two-way ANIs of Mbale, Mbale2, and Mbale3 to the reference strain were 97.05%, 97.03%, and 97.01%, respectively, which fall above 95% sequence similarity threshold defining species (26). Biochemical testing using API test strips read at 48 h also identified the reference, Mbale, Mbale2, and Mbale3 strains as *P. thiaminolyticus* at 99.9%, 99.9%, 97 to 98.6%, and 91.6 to 98.9% confidence (Table S1).

10.1128/mbio.02688-22.6 TABLE S1 Results of biochemical testing of all four isolates. Download Table S1, DOCX file, 0.02 MB. Copyright © 2022 Hehnly et al. 2022 Hehnly et al. https://creativecommons.org/licenses/by/4.0/ This content is distributed under the terms of the Creative Commons Attribution 4.0 International license.

### Genomic comparisons of *P. thiaminolyticus* reference strain and clinical isolates.

Multiple-sequence alignment (27) identified 12 locally colinear blocks between the four strains, representing sequences with conserved segments and no rearrangements (Fig. 1C). The genomes also contained several regions of low sequence similarity. The contigs that did not assemble into the chromosome had no homology to the reference strain. The number and classes of proteins predicted to be encoded by each of the three clinical isolates and the reference strain, as determined by RefSeq (28) or PATRIC (29), are listed in Table 1. Consistent with the high similarity of the genomes of the clinical isolates and reference strain, these genomes carry very similar sets of metabolic genes and, accordingly, are predicted to encode similar metabolic pathways (Table 2). Many of the predicted carbohydrate metabolic pathways were confirmed by biochemical testing (Table S1). The clinical isolates and reference strain all carry several antibiotic resistance genes and encode a thiol-activated cytolysin, a potential virulence factor. The reference strain carries an operon encoding a putative type VII secretion system, which the clinical isolates lack.

**TABLE 2:** Metabolic pathways common to clinical and reference strains

Pathway and subsystem	No. of subsystems (no. of genes) in:			
	B-4156	Mbale	Mbale2	Mbale3
Metabolism	91 (745)	93 (767)	92 (742)	94 (768)
Cofactors, vitamins, and prosthetic groups	21 (228)	22 (246)	20 (231)	21 (227)
Amino acids and derivatives	29 (227)	29 (225)	29 (220)	29 (241)
Fatty acids, lipids, and isoprenoids	11 (88)	11 (91)	11 (88)	11 (92)
Carbohydrates	9 (64)	8 (65)	9 (60)	9 (69)
Nucleosides and nucleotides	8 (64)	9 (62)	8 (62)	4 (27)
Iron acquisition and metabolism	4 (25)	4 (25)	4 (28)	4 (17)
Phosphate metabolism	1 (20)	2 (21)	2 (12)	2 (15)
Metabolite damage and mitigation	4 (17)	4 (17)	4 (25)	2 (7)
Secondary metabolism	2 (7)	2 (10)	2 (10)	2 (5)
Nitrogen metabolism	1 (4)	1 (4)	2 (5)	2 (5)
Sulfur metabolism	1 (1)	1 (1)	1 (1)	1 (1)
Protein processing	44 (249)	43 (255)	42 (237)	41 (225)
Protein synthesis	31 (201)	31 (203)	30 (188)	29 (177)
Protein fate	13 (48)	12 (52)	12 (49)	12 (48)
Energy	28 (205)	29 (218)	27 (209)	29 (235)
Energy and precursor metabolite generation	18 (124)	20 (141)	18 (134)	20 (159)
Respiration	10 (81)	9 (77)	9 (75)	9 (76)
Cellular processes	27 (174)	27 (183)	27 (175)	26 (175)
Cell cycle, cell division, and death	11 (82)	11 (82)	13 (82)	11 (83)
Prokaryotic cell differentiation	13 (77)	13 (83)	11 (78)	12 (77)
Mobility and chemotaxis	1 (9)	1 (9)	1 (9)	1 (9)
Clustering-based subsystems	1 (5)	1 (8)	1 (5)	1 (5)
Microbial communities	1 (1)	1 (1)	1 (1)	1 (1)
Stress response, defense, virulence	36 (120)	17 (119)	32 (121)	31 (124)
Resistance to antibiotics and toxic compounds	19 (66)	17 (61)	15 (65)	15 (124)
Stress response: heat/cold	2 (17)	2 (22)	2 (18)	2 (17)
Stress response: osmotic stress	4 (13)	7 (13)	4 (13)	7 (15)
Stress response: electrophile toxicity	1 (4)	1 (4)	1 (4)	1 (4)
Host-pathogen interactions	11 (72)	1 (1)	1 (1)	2 (6)
DNA processing	18 (89)	19 (117)	18 (90)	19 (116)
DNA repair	12 (59)	13 (96)	12 (60)	13 (84)
DNA uptake, competence	2 (16)	2 (28)	2 (15)	2 (16)
DNA recombination	2 (5)	1 (4)	2 (6)	2 (5)
DNA replication	1 (4)	1 (2)	1 (4)	1 (5)
Membrane transport	11 (72)	11 (68)	7 (63)	7 (68)
ABC transporters	1 (42)	1 (41)	1 (42)	1 (41)
Cation transporters	4 (22)	3 (17)	3 (17)	3 (21)
Multidrug efflux systems	2 (3)	2 (3)	2 (3)	2 (4)
Protein secretion systems, type VII	3 (4)	0 (0)	0 (0)	0 (0)
Protein secretion systems, type II	1 (1)	1 (2)	1 (1)	1 (2)
Uniporter, Symporter, Antiporter	0 (0)	1 (1)	0 (0)	0 (0)
RNA processing	12 (52)	12 (54)	12 (51)	2 (16)
Cell envelope	3 (15)	3 (15)	3 (14)	1 (6)
Regulation and cell signaling	3 (12)	3 (12)	3 (14)	3 (14)

**FIG 2:**
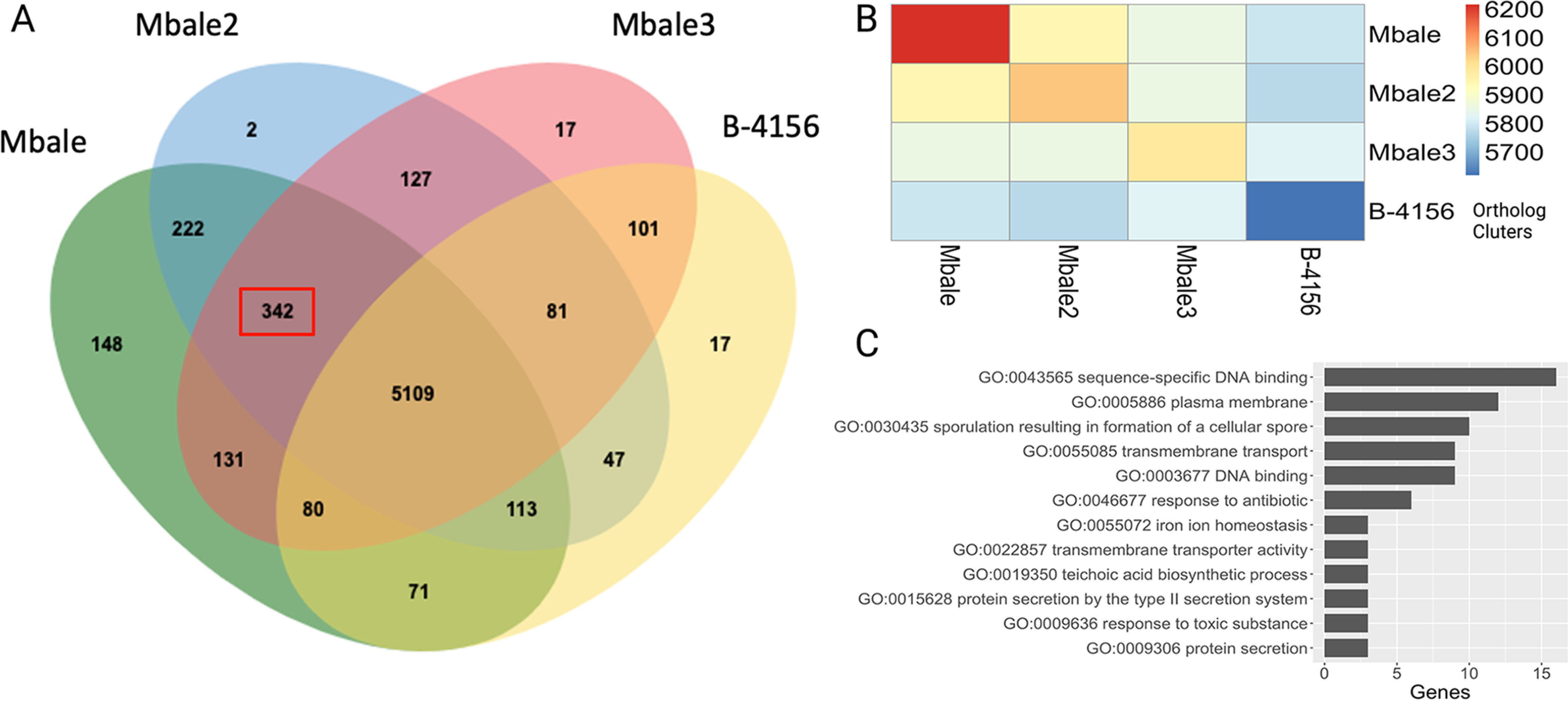
Comparisons of the predicted proteomes of the clinical isolates and the reference strain. (A) Venn diagram from OrthoVenn2 analysis comparing the annotations of the B-4156, Mbale, Mbale2, and Mbale3 strains. The 342 orthologous clusters among the clinical isolates that were absent in the nonpathogenic B-4156 are outlined in red. (B) Heat map quantifying the number of predicted orthologous clusters across each isolate. (C) Clinically relevant gene ontology terms from the 342 proteins that were unique to the clinical isolates.

As a means of identifying potentially clinically relevant features of the clinical strains, we used OrthoVenn2 and a function-based comparison in RASTtk. OrthoVenn2 identified 6,608 unique clusters of orthologous protein across all four strains, with 5,109 clusters present in all isolates (Fig. 2A). Overall, the clinical strains share more coding regions among themselves than with the reference strain (Fig. 2B). Specifically, the three clinical isolates share 342 orthologous clusters that are absent from the reference strain. Gene ontology analysis of these 342 coding regions returned 39 terms, with the largest number of genes associated with “sequence-specific DNA binding,” “plasma membrane,” and “sporulation” (Table S2). Several genes populate terms such as “secretion,” “iron,” and “response to toxins,” which could contribute to the difference in virulence between the isolates and the reference strain (Fig. 2C).

10.1128/mbio.02688-22.7 TABLE S2 Gene ontology terms associated with 342 unique orthologous clusters in clinical isolates. Download Table S2, DOCX file, 0.01 MB. Copyright © 2022 Hehnly et al. 2022 Hehnly et al. https://creativecommons.org/licenses/by/4.0/ This content is distributed under the terms of the Creative Commons Attribution 4.0 International license.

A function-based comparison between each clinical isolate and the reference strain in RASTtk uncovered unique coding regions (Table S3), eight of which were shared across all three clinical isolates (Table 3). This revealed that all three isolates contained an insert carrying a 3-gene operon for the biosynthesis of 2-aminoethylphosphonate. 2-Aminoethylphosphonate is produced by a variety of bacteria and protists (30) and has been shown to decorate the surface of Clostridioides difficile (31). On the other hand, this pathway could also catalyze utilization of 2-aminoethylphosphonate as a source of carbon, phosphorous, and nitrogen. The strains also carry stand-alone genes coding for predicted proteins involved in tryptophan and teichoic acid processing. Mbale and Mbale3, but not the reference strain or Mbale2, shared the genes of the *citSDEFCXG* operon, which encodes a Na^+^/citrate symporter, three subunits of citrate lyase (EC 4.1.3.6), and the enzymes responsible for the synthesis and attachment of the coenzyme A (CoA)-like prosthetic group of citrate lyase (32). The presence of a functional citrate lyase could contribute to bacterial survival under hypoxic conditions (33, 34). These unique predicted proteins (listed in Table 3) were all confirmed in the Mbale strain by proteomic analysis (see below).

**TABLE 3:** Predicted functional proteins in the clinical isolates but absent in reference strain B-4156

Category	Predicted function
Secondary metabolism	Phosphoribosylanthranilate isomerase (EC 5.3.1.24)
Phosphonate metabolism	Phosphoenolpyruvate phosphomutase (EC 5.4.2.9)
	Phosphoenolpyruvate decarboxylase (EC 4.1.1.82)
	2-Aminoethylphosphonate: pyruvate aminotransferase (EC 2.6.1.37)
Membrane transport and secretion systems	Leader peptidase (prepilin peptidase) (EC 3.4.23.43)
	Type IV fimbrial assembly protein PilC
	Twitching motility protein PilT
Cell wall and capsule	Teichoic acid translocation permease protein TagG

10.1128/mbio.02688-22.8 TABLE S3 Predicted functional proteins unique to the clinical isolates. Download Table S3, DOCX file, 0.02 MB. Copyright © 2022 Hehnly et al. 2022 Hehnly et al. https://creativecommons.org/licenses/by/4.0/ This content is distributed under the terms of the Creative Commons Attribution 4.0 International license.

### Mobile genetic element prediction and annotation.

**FIG 3:**
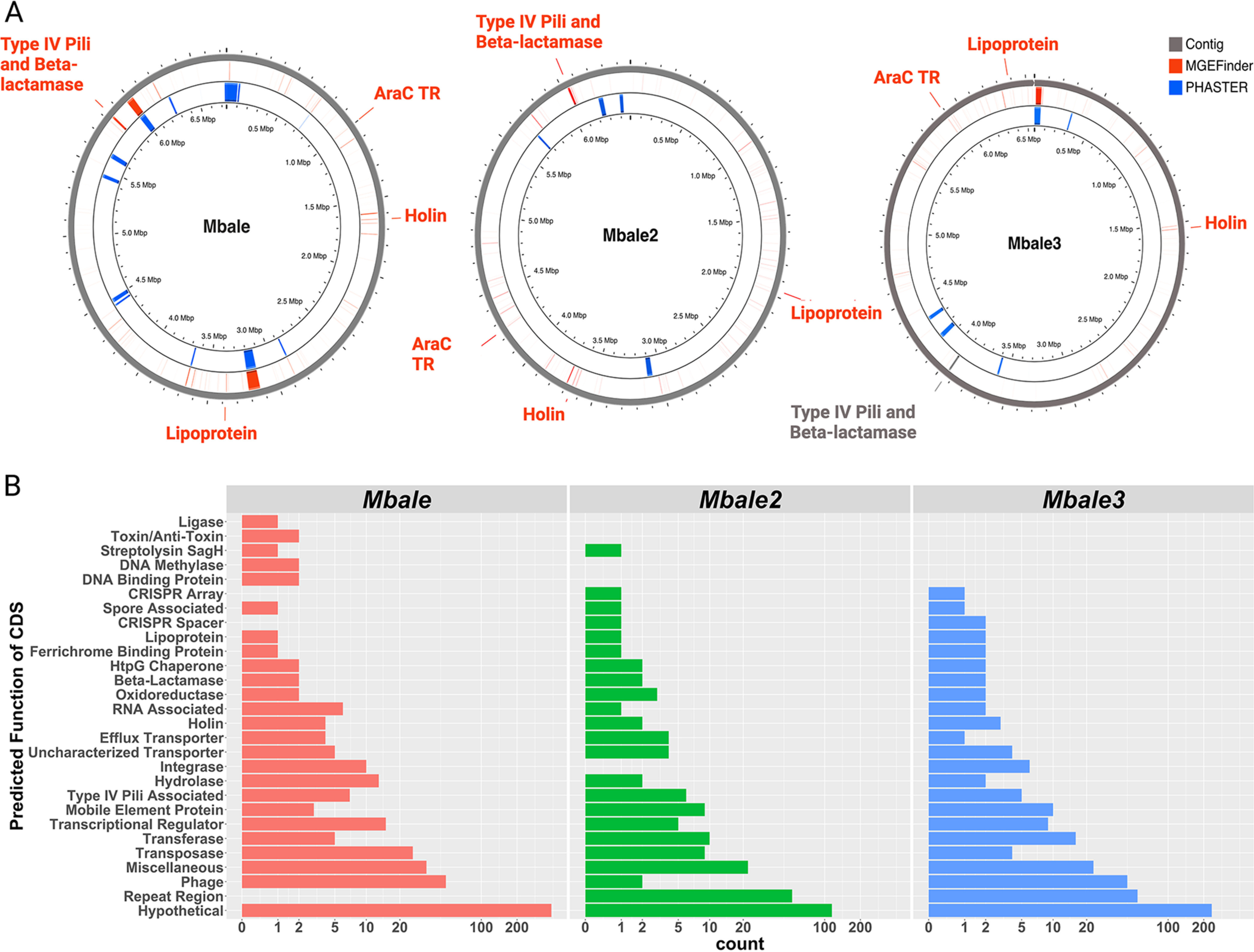
Mobile genetic element identification and annotation. (A) MGEfinder identified regions of genomes that could have been derived from MGEs (red bars) and could account for the regions of low sequence similarity. Genes specified in red were identified by RAST as being encoded in the predicted MGEs and include a lipoprotein, holin, and an arabinose family transcription regulator (AraC TR). A predicted MGE in Mbale and Mbale2 included an operon for a putative type IV pilus, which, although not predicted to be an MGE in Mbale3, was also present in that genome (gray). Regions of phage sequences, identified by PHASTER, are labeled in blue. (B) Bar plot of the predicted functions from the RAST annotation of coding sequences that aligned to the MGEfinder-predicted inserts.

Horizontal transfer of mobile genetic elements (MGEs) facilitates rapid evolution of microbial genomes (35, 36). MGEfinder (37) identified 320 sequences (186 unique) with 99% sequence identity across all three clinical isolates. The 320 sequence insertions range from 70 to 82,113 bp in length (median, 168 bp; interquartile range, 112 to 352 bp). Mbale, Mbale2, and Mbale3 were found to carry 158, 156, and 129 inserted sequences, respectively, as well as 11, 4, and 5 phage insertions (Fig. 3A).

To assess the possible functional significance of the insertion sequences predicted by the MGEfinder, we annotated them with RAST (38) and performed functional enrichment analysis with STRING (39). This analysis yielded 676 predicted coding sequences associated with 22 gene ontology terms (Fig. S2), dominated by genes associated with DNA processing and the establishment of competence.

10.1128/mbio.02688-22.2 FIG S2 Gene ontology terms of annotated insertions from MGEfinder. (A) Bar plot of the numbers of genes identified to each gene ontology terms. (B) STRING analysis of the 342 unique clinical isolate proteins identified a network of protein interactions between uncharacterized proteins (YqaK, YgaS, YbcM, YqaT, YqbK, and YqbT) and phage terminase (XtmA), along with additional protein-protein interactions related to competence (ComC and ComA), DNA repair (RecA, PcrA, and DnaC), and protein transport (YxlE and YxlF). Download FIG S2, DOCX file, 0.5 MB. Copyright © 2022 Hehnly et al. 2022 Hehnly et al. https://creativecommons.org/licenses/by/4.0/ This content is distributed under the terms of the Creative Commons Attribution 4.0 International license.

Finally, to evaluate the extent to which difference between the reference strain and the clinical isolates derived from MGE inserts, we aligned the 186 unique predicted inserts from MGEfinder to the RASTtk-predicted protein sequences from each clinical isolate using BLAST (96% similarity). Mobile genetic elements encompassed 515, 201, and 331 coding sequences in Mbale, Mbale2, and Mbale3, respectively. Of the orthologs identified in the clinical strains, 22% (77/355), 18% (64/346), and 17% (58/348), respectively, were encoded in these insertion sequences, indicating that a substantial portion of the variability from the reference strain that overlaps in all the clinical isolates results from MGEs. Grouped based on their predicted function, most of the MGE-encoded proteins were hypothetical, phage related, or repeat regions (Fig. 3B). Besides these, all three clinical isolates contained 79 genes that could contribute to virulence, including those encoding a unique AraC family transcriptional regulator, a holin, and a putative lipoprotein (Fig. 3A). In addition, Mbale, Mbale2, and Mbale3 carried a common 12-kbp insert spanning a predicted type IV pilus (T4P) operon followed by a gene that codes for a beta-lactamase class C-like protein (Fig. 3A). Consistent with the presence of a potential beta-lactamase gene, two of the three isolates were resistant to the beta-lactam antibiotics ampicillin and penicillin (Table S4).

10.1128/mbio.02688-22.9 TABLE S4 Antibiotic susceptibility testing results of the three clinical isolates and the reference strain. Download Table S4, DOCX file, 0.01 MB. Copyright © 2022 Hehnly et al. 2022 Hehnly et al. https://creativecommons.org/licenses/by/4.0/ This content is distributed under the terms of the Creative Commons Attribution 4.0 International license.

### RNA and protein expression analysis of Mbale and the reference strain.

**FIG 4:**
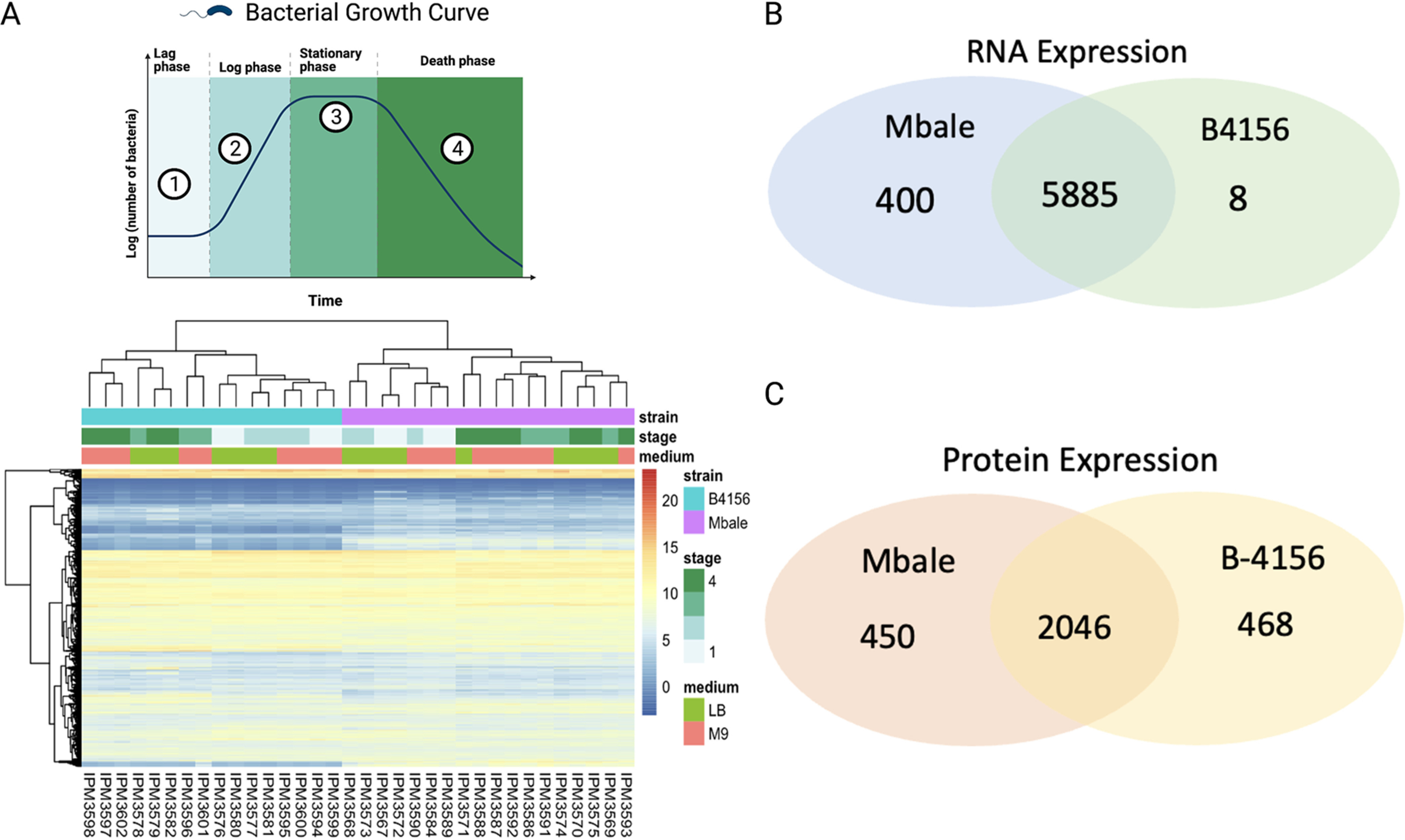
RNA and protein expression of the B-4156 and Mbale strains. (A) Culture growth stages (top) and unsupervised hierarchical clustering (bottom) of the RNA transcript levels of the most variable genes expressed in Mbale and the reference strain of *P. thiaminolyticus*. Clusters separate primarily according to strains and secondarily on the basis of stage of growth and media (B and C) Comparison of RNA (B) and protein (C) expression between Mbale and B-4156.

To assess whether the predicted coding regions are transcribed, we performed sequence analysis of RNA isolated from Mbale and reference strains grown in two different media, LB and minimal salts (M9) plus glucose, at four stages of growth (lag phase, middle log phase, late log phase, stationary phase, and death phase) (Fig. 4A). We identified 6,285 and 5,893 unique transcripts from the Mbale and reference strains, respectively, with 5,885 transcripts present in both strains, 400 unique to Mbale and 8 unique to the reference strain (Fig. 4C). Unsupervised hierarchical clustering of the most variable expressed transcripts provided clear discrimination based on strain and stage of growth, with some distinction related to growth media (Fig. 4A).

To assess the translation of predicted proteins, we performed proteomics analysis of the reference and Mbale strains growing exponentially in LB medium. This analysis identified 193,878 and 194,234 peptides in the Mbale and reference strains, respectively, which mapped to 2,488 and 2,531 proteins based on the annotation by NCBI’s Prokaryotic Genome Annotation Pipeline (PGAP) (28). Two thousand forty-six proteins were present in both strains, while 450 unique proteins were expressed only in Mbale and 468 only in the reference strain (Fig. 4C). Gene ontology analysis (40) of the unique proteins in each strain confirmed the presence of proteins from similar metabolic pathways despite the unique protein sequences. Two unique terms, “Bacterial secretion system” and “Protein export,” were ascribed to the Mbale strain (Fig. S3). The genes assigned to these terms code for the SecA secretion pathway (*secY*, *secA*, *ffH*, *laspA*, *secF*, and *secD*), which are distinct from the genes in the T4P operon. In addition, the *pilC*- and *tagG*-encoded predicted proteins unique to Mbale were confirmed.

10.1128/mbio.02688-22.3 FIG S3 Gene ontology of the unique proteins in each isolate. Gene ontology of the 450 proteins uniquely identified in proteomic data for the B-4156 strain (left) and the 468 proteins unique to the Mbale strain (right). *P* values indicate the extent of enrichment of the genes of each indicated ontological group in the set of genes unique to each of the strains. Bacterial secretion system and protein export genes related to the SecA pathway uniquely represented in the Mbale strain are highlighted in red. Download FIG S3, DOCX file, 0.5 MB. Copyright © 2022 Hehnly et al. 2022 Hehnly et al. https://creativecommons.org/licenses/by/4.0/ This content is distributed under the terms of the Creative Commons Attribution 4.0 International license.

### The Mbale T4P system is a virulence factor.

**FIG 5:**
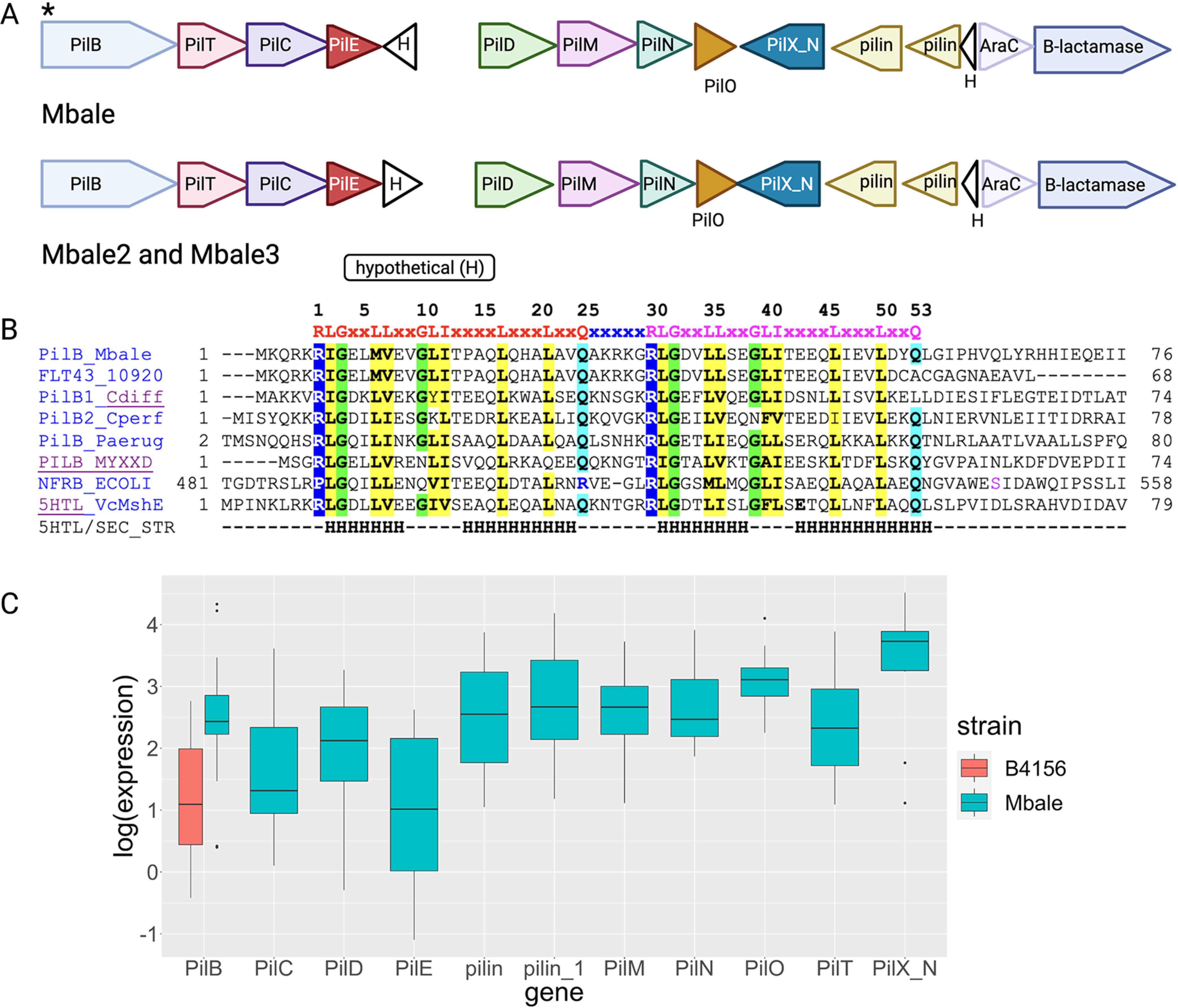
Predicted insertion carries the full Gram-positive T4P operon. (A) The T4P operon present in all three clinical isolates and absent in strain B-4156 is located in the predicted mobile genetic element insertion (in strain Mbale, locus tags FLT15_06255 to FLT15_06190). The genes were annotated with RAST or PGAP and/or with hits in the COG and Pfam databases. PilFind (42) was used to identify potential pilins (light brown). White triangles designate hypothetical proteins of unknown function. The asterisk indicates a c-di-GMP-binding site in PilB. (B) Sequence alignment of the N-terminal fragment of PilB from strain Mbale (GenBank accession number NGP58005.1) and truncated protein FLT43_10920 (GenBank number QDM43956.1) from *P. thiaminolyticus* reference strain B-4156 against experimentally characterized MshEN domains. The top line shows the conserved c-di-GMP-binding site of the MshEN domain, which consists of tandem 24-amino-acid (aa) motifs separated by a 5-aa insert (45). Aligned sequences include MshEN domains of PilB proteins from Clostridioides difficile (47), Clostridium perfringens (46), Pseudomonas aeruginosa (PA3740) (45, 91), and Myxococcus xanthus (MXAN_5788) (92) and of Escherichia coli NfrB (93, 94). The bottom two lines show the sequence and secondary structure (H, α-helix) of the structurally characterized MshEN protein from Vibrio cholerae (VC_0405) (44, 45). Conserved hydrophobic residues are shaded yellow, and conserved Gly residues are shaded green. (C) Expression levels of the *pil* genes obtained from the transcriptome sequencing (RNA-seq) data.

The alignment of the predicted insertions from MGEfinder to the initial annotation identified a 12- to 14-kbp insertion encoding T4P components in all three clinical isolates. Further analysis with protein family-specific profiles and pilFind (41, 42) confirmed that all the structural and assembly components requires for a type 4 pilus were encoded in the operon within a mobile element in all three strains (Fig. 5A; Table 4). Comparison of the gene order in the 14-kbp insertion of *P. thiaminolyticus* Mbale with the previously characterized *pil* operons from other Gram-positive bacteria (42, 43) showed the closest match to the *pil* operons of Clostridium cellulolyticum H10 and *Bacillus* sp. strain NRRL B-14911 (Fig. S3). The Mbale2 and Mbale3 insertion sequences contained the same *pil* genes as the Mbale sequence (Fig. 5A). In contrast, the reference strain has only a truncated *pilB* gene (encoding the first 60 amino acids of PilB) and lacks the remaining genes required for the assembly of the T4P. The T4P assembly ATPase, PilB, of strain Mbale contains an N-terminal MshEN domain with a perfectly conserved motif for binding the bacterial second messenger cyclic di-GMP (c-di-GMP) (44, 45) (Fig. 5B). Similar MshEN domains are found at the N termini of PilB-like ATPases in several bacterial pathogens, including Pseudomonas aeruginosa, Clostridium perfringens and Clostridioides difficile (Fig. 5B), and c-di-GMP binding by these ATPases has been shown to regulate T4P production (45–47). Accordingly, regulation of T4P production by c-di-GMP can also be expected to take place in clinical strains of *P. thiaminolyticus*. All genes in the T4P cluster are transcribed in the Mbale strain, as is the MshEN-encoding *pilB* fragment in the reference strain (Fig. 5C).

**TABLE 4:** Predicted type IV pilus proteins in Paenibacillus thiaminolyticus Mbale^*a*^

Protein function	Protein domain (COG, pfam)	Type II secretion protein in:		Type IV pilus protein (gene name or accession no.) in:			
		Escherichia coli K-12	Klebsiella oxytoca	Pseudomonas aeruginosa	Clostridium perfringens	Streptococcus sanguis 2908	Paenibacillus thiaminolyticus Mbale1
Pilus assembly ATPase	COG2804, PF00437	GspE	PulE	PilB (PA4526)	PilB (CPE1844, CPE2286)	PilF (SSV_2244, CEL91512.1)	FLT15_06255 (NGP58005.1) (566 aa)
Pilus retraction ATPase	COG2805, PF00437	NP	NP	PilT/PilU (PA0395, PA0396)	PilT (CPE1767)	PilT (SSV_2243, CEL91511.1)	FLT15_06250 (NGP58004.1) (362 aa)
Core membrane protein	COG1459, PF00482	GspF	PulF	PilC (PA4527)	PilC (CPE2285, CPE1843)	PilG (SSV_2242, CEL91510.1)	FLT15_06245 (NGP58003.1) (403 aa)
Prepilin/pseudopilin	COG2165, PF07963, COG4968	GspG, GspH, GspI, GspJ, GspK	PulG	PilE/PilV/PilW (PA4551, PA4552/56)	PilE (CPE2284)	PliE1 (SSV_2241, CEL91509.1), PilE2 (SSV_2240, CEL91508.1)	FLT15_06240 (NGP58002.1) (142 aa)
Prepilin peptidase	COG1989	GspO	PulO	PilD (PA4528)	PilD (CPE2287)	PilD (SSV_2230, CEL91498.1)	FLT15_06230 (NGP58000.1) (255 aa)
Pilus alignment protein PilM	COG4972	GspL	PulL	PilM (PA5044)	PilM (CPE2283)	PilM (SSV_2236, CEL91504.1)	FLT15_06225 (NGP57999.1) (387 aa)
Pilus alignment protein PilN	PF05137	GspM	PulM	PilN (PA5045)	PilN (CPE2282)	PilN (SSV_2235, CEL91503.1)	FLT15_06220 (NGP57998.1) (200 aa)
Pilus alignment protein PilO	COG3167, PF04350	NP	NP	PilO (PA5046)	PilO (CPE2281, CPE2288)	PilH (SSV_2234, CEL91502.1)	FLT15_06215 (NGP57997.1) (252 aa)
Major pilin/pseudopilin	COG4969, PF00114	GspG	PulG	PilA (PA4525)	PilA (CPE2288)	PilA (SSV_2239, CEL91507.1)	FLT15_06210 (NGP57996.1) (475 aa)

a The table shows protein families that include main components of the type IV pili and type II secretion systems (T2SS) and their gene names in selected Gram-negative and Gram-positive species. The table is based on the reviews by Melville and Craig (41) and Pelicic (60). The protein domain data are from COG, Pfam, and InterPro databases (95–97). Type II secretion data are from references 98–100, and T4P data are from reference 101 for P. aeruginosa and from reference 102 for C. perfringens. Protein assignments for *P. thiaminolyticus* Mbale are based on the results of BLAST searches and comparisons against COG, Pfam, and InterPro databases. The *P. thiaminolyticus* reference strain B-4156 encodes only the N-terminal 60-aa fragment of PilB and none of the other proteins. Note the lack of the pilus retraction ATPase PilT in the T2SS and nonstandard gene names for PilB, PilC, and PilO in *S. sanguis*. NP, not present.

**FIG 6:**
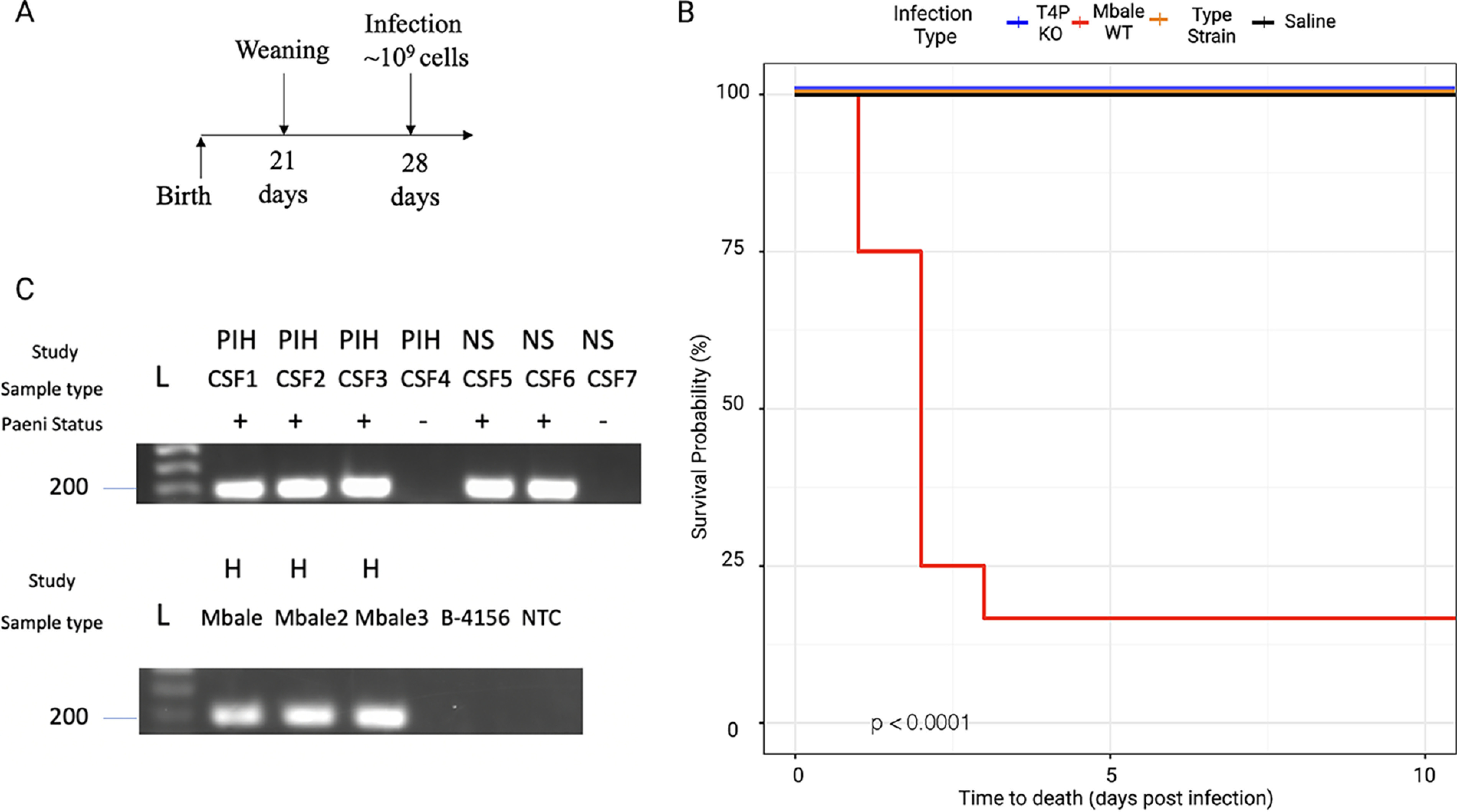
The T4P is a critical virulence factor in all three clinical isolates. (A) Mouse sepsis model of infection with injection performed postweaning on day 28 of life. (B) Kaplan-Meier survival curve depicting survival of mice following injection of the T4P deletion strain (*n* = 9; blue), the wild-type Mbale strain (*n* = 9; red), the reference strain B-4156 (*n* = 9; orange), or vehicle control (*n* = 4; black) (*P* < 0.0001). (C) Amplification of the *pilT* gene in CSF or bacterial culture (H) from infants with postinfectious hydrocephalus (PIH) due to *P. thiaminolyticus* or neonatal sepsis (NS) due to *P. thiaminolyticus* that subsequently led to PIH. *Paenibacillus* status (Paeni status) was determined by qPCR of the previously defined thiaminase gene and was 100% concordant with *pilT* positivity (22). L, DNA ladder.

Previously, we established a mouse model of neonatal sepsis to demonstrate the differential virulence of the Mbale and reference strains (13). To directly test the role of the T4P in virulence, we used CRISPR-Cas9 to construct a mutant of the Mbale strain from which the *pilB*, *pilC*, and *pilT* genes were deleted. As described in Materials and Methods, we constructed plasmid pAS3 carrying a mannose-inducible-promoter controlled Cas9 expression, a single guide RNA (sgRNA) targeting the region adjacent to the *pilC* gene, and a homology-directed recombination template that would yield a deletion of the three targeted genes (Fig. S4 and S5). Following the introduction and propagation of the plasmid in Mbale, we recovered several colonies from which the three genes were deleted. We assessed the virulence of one of the mutant strains in a mouse infection model (Fig. 6A) and determined that, with identical bacterial doses, none (0/9) of the Mbale-injected mice survived while all the mice injected with the deletion (9/9) or reference (9/9) strain survived (Fig. 6B) (*P* < 0.0001). This result confirms that the T4P system in the Mbale strain contributes to its pathogenesis. To confirm that the decrease in virulence phenotype was specific to the T4P deletion, we performed whole-genome sequencing (WGS) of the knockout strain. Using the Breseq variant pipeline with 20× coverage of the Mbale genome and PCR confirmation, we determined that no off-target deletions were present in the T4P knockout.

10.1128/mbio.02688-22.4 FIG S4 Comparison between the previously identified T4P operon and the Mbale clinical isolate. Comparison of the gene order in the *pil* operon of *P. thiaminolyticus* Mbale with the previously characterized *pil* operons from other Gram-positive bacteria showed the closest match to the *pil* operons of Clostridium cellulolyticum H10 and *Bacillus* sp. strain NRRL B-14911. The latter strain was originally isolated from the Gulf of Mexico but was subsequently reclassified as Bacillus infantis; its genome (GenBank accession number CP006643.1) is currently the only complete genome sequence from that species. Remarkably, *B. infantis* was so named after being isolated from a case of neonatal sepsis in Busan, South Korea. Although not recognized at the time, T4P could have contributed to virulence in that case as well. Download FIG S4, DOCX file, 0.4 MB. Copyright © 2022 Hehnly et al. 2022 Hehnly et al. https://creativecommons.org/licenses/by/4.0/ This content is distributed under the terms of the Creative Commons Attribution 4.0 International license.

10.1128/mbio.02688-22.5 FIG S5 Validation and schema of T4P knockout in the Mbale isolate. (A) PCR products were run on a 1% agarose gel spanning the *pilC*, *pilT*, and *pilB* genes. Comparing the sizes of the PCR products of the wild type (WT) to the knockout strain (KO) identifies a 4,500-bp knockout. (B) Schematic including the guide RNA (sgRNA; red) for the CRISPR-Cas9 cut in the genome and primers with appropriate locations in the genome that were used for the PCR confirmation. Download FIG S5, DOCX file, 1.2 MB. Copyright © 2022 Hehnly et al. 2022 Hehnly et al. https://creativecommons.org/licenses/by/4.0/ This content is distributed under the terms of the Creative Commons Attribution 4.0 International license.

### *pilT* may serve as a diagnostic marker for *P. thiaminolyticus* infections.

We previously identified *P. thiaminolyticus* in the CSF of a subset of patients with PIH or neonatal sepsis using qPCR to the thiaminase gene (22). To determine whether the T4P is associated with infection, we used PCR to probe for the *pilT* gene in clinical samples previously determined to be positive or negative for *Paenibacillus*. As a control, we probed for the *pilT* gene in all three clinical isolates and reference strain and showed that we could detect it in the clinical strains but not in the reference strain (Fig. 6C). Examining clinical samples, we detected *pilT* in the CSF from all three PIH cases with positive *P. thiaminolyticus* cultures and two neonates with sepsis who subsequently developed PIH with *P. thiaminolyticus* infections. We did not detect *pilT* in several CSF samples previously determined to be *Paenibacillus* negative (Fig. 6C). These results suggest that the presence of *pilT* correlates with infection by *Paenibacillus*, consistent with a role for the T4P cluster in pathogenesis. Moreover, these results suggest that *Paenibacillus* with *pilT* may be detectable in clinical samples prior to the onset of hydrocephalus.

## DISCUSSION

### Pathogenicity of Paenibacillus thiaminolyticus.

*Paenibacillus* species have been isolated and studied from various sources, particularly in agricultural and industrial settings. These rod-shaped, Gram-positive, endospore-forming facultative anaerobic bacteria were initially assigned to the genus *Bacillus* but were subsequently recognized as being substantially distinct from other *Bacillus* spp. and assigned to a separate genus, *Paenibacillus* (“almost bacillus”), with Paenibacillus polymyxa as the type species (48).

We recently identified *P. thiaminolyticus* as a novel pathogen associated with postinfectious hydrocephalus and neonatal sepsis in Ugandan infants (13). In this work, sequencing, assembly, and functional annotation of the genomes of three clinical isolates and the reference strain of *P. thiaminolyticus* provided a comprehensive catalog of the genetic content of these strains, and our transcriptomic and proteomic analysis confirmed expression of a significant fraction of the predicted genes (Table 2; Fig. 3). While the clinical isolates exhibit more than 97% identity with the reference strain (Fig. 1B), they differ from the reference strain predominantly by the presence of many insertions, likely derived by horizontal transfer of mobile genetic elements (Fig. 1C). These insertions are predicted to encode mobile element proteins, hypothetical proteins, multiple AraC family transcriptional regulators, and phage proteins. The AraC family of transcriptional regulators has been implicated in regulation of proteins with diverse functions, including virulence factors (49, 50). In addition, a unique holin and a putative lipoprotein were identified in the clinical isolates. Holins have been shown to be important for toxin secretion in Clostridium perfringens (51). Lipoproteins have been shown to be integral in the pathogenicity of various pathogens, contributing to adhesion, immune evasion, and translocation of virulence factors into the host (52). In a previous report, we identified host upregulation of Toll-like receptor 2 or 4 and cofactor CD14, which are known to recognize lipoproteins (53), during a *Paenibacillus* infection in the central nervous system of infants with PIH (54). Finally, all three characterized isolates carry a unique beta-lactamase gene, and two of the three isolates exhibit resistance in culture to the beta-lactam antibiotics penicillin and ampicillin. Since the first-line antibiotic regimen recommended by the World Health Organization for neonatal sepsis is ampicillin and gentamicin, our findings indicate that updated guidelines should be considered for the infections due to this organism (55).

### Type IV pili as a virulence factor.

Of particular interest regarding virulence of the clinical isolates, all three strains carry a 12- to 14-kbp insertion with the genes necessary for the assembly of type IV pili (T4P), linked to the genes for an AraC transcription regulator and an AmpC-type beta-lactamase-like protein (Fig. 3A). Proteomic analysis of the Mbale strain documented expression of genes in this operon (Table 3). Significantly, deletion of several genes within the operon substantially reduced the virulence of the Mbale strain in the mouse infection model. This strongly suggests that T4P contribute to the pathogenesis of the clinical isolates.

T4P are thin appendages present in numerous bacterial pathogens, which have been implicated in an array of functions, including cellular adhesion, cell mobility, protein secretion, biofilm formation, and DNA uptake (reviewed in references 41 and 56–58). The T4P were initially observed exclusively in Gram-negative bacteria, but with the advent of whole-genome sequencing, many conserved components of the T4P have been identified and studied in various Gram-positive species (41, 42, 59–61). T4P in Gram-positive organisms include the same core components as the better-studied T4P from Pseudomonas aeruginosa, Myxococcus xanthus, and other Gram-negative bacteria, with the exception of the periplasmic lipoprotein PilP, the pilotin PilF, and the outer membrane secretin PilQ (62–64). In addition to the major pilin (PilA) and occasional minor pilins, these components include two hexameric ATPases of the AAA+ family, the pilus assembly (extension) ATPase PilB and the pilus retraction (depolymerization) ATPase PilT, as well as the inner membrane protein PilC, the prepilin peptidase PilD, and the pilus alignment (stator) components PilM, PilN, and PilO (41, 63). The T4P assembly complex is very similar to the type II secretion system (T2SS), which, however, does not include the pilus retraction ATPase PilT (41); pilus retraction in T2SS and in certain T4P systems appears to occur spontaneously (65). Thus, presence of the *pilT* genes in all three Mbale strains indicates that these operons code for T4P and not T2SS.

The T4P gene cluster in clinical isolates of *P. thiaminolyticus* is closely related to those in Clostridium cellulolyticum H10 and *Bacillus* sp. strain NRRL B-14911 (Fig. S4). The latter strain was originally isolated from the Gulf of Mexico (66) but was subsequently reclassified as Bacillus infantis (67). Notably, the reference strain of *B. infantis* was isolated from a case of neonatal sepsis in Busan, South Korea (68), an observation that reinforces the possible connection between T4P and neonatal sepsis. The T4P operons of Mbale strains are also similar to those from C. perfringens and C. difficile; in all of them, PilB ATPases contain a c-di-GMP-binding MshEN domain (Fig. 5B). In C. perfringens, the formation of T4P is controlled by c-di-GMP (46), which suggests that the same might be true for the clinical isolates of *P. thiaminolyticus.* In C. difficile, c-di-GMP-stimulated production of T4P promoted adherence to epithelial cells, cell aggregation, and enhanced biofilm formation (47, 69, 70). The potential regulation of T4P expression by c-di-GMP may account for our anecdotal observation that cells of strain Mbale appear to have higher virulence after growth on agar plates than after growth in liquid culture (data not shown). While we have not rigorously quantified this observation, it is consistent with the reported higher T4P production in surface-grown C. perfringens (71). A detailed analysis of the regulation of T4P expression in the Mbale strains is expected to provide further insights into the potential contribution of c-di-GMP and biofilm formation to the virulence of the clinical isolates of *P. thiaminolyticus*.

T4P is a widespread virulence factor that is structurally conserved, which makes it an attractive diagnostic and therapeutic target. Previously, Barnier et al. (72) showed that the T4P-mediated twitching motility by the PilT protein in the Gram-negative pathogen Neisseria meningitidis is required for sustained bacteremia. Moreover, they showed that adjunctive treatment with phenothiazines targeting T4P reduced vascular colonization and associated inflammation in a mouse model (73). The impact of the phenothiazine family of drugs on the Gram-positive T4P warrants further investigation and could provide a valuable adjunctive therapeutic approach to the treatment of neonatal sepsis and PIH caused by *P. thiaminolyticus*.

Successful pathogenic bacteria must propagate and survive within a host, which likely requires more than just one virulence factor. By identifying differences in the clinical strains, we have uncovered several candidate genes that may be involved in pathogenesis (Fig. 2 and 3; Table 3). However, focusing only on differences between the clinical isolates and reference strain may miss certain virulence factors, such as toxins, that could be present in both pathogenic and nonpathogenic bacteria but mobilized only in the former due to the inability of nonpathogenic bacteria to survive in the host or to deliver the toxin (74, 75). For instance, our clinical isolates as well as the reference strain encode a thiol-dependent cytolysin with 84% similarity to alveolysin, the toxin in Paenibacillus alvei that can lyse and inactivate eukaryotic cells (76–78). Further functional genetic analysis of the clinical isolates will be required to identify additional functional virulence factors. Regardless, we demonstrated that comparative proteogenomics of differentially virulent bacteria of the same species could identify critical virulence factors that have not been identified from the automated genome annotation alone. Specifically, we pinpointed the T4P genes as critical for virulence in the clinical isolates of *P. thiaminolyticus* associated with PIH and NS and showed that they were acquired via a common MGE. This methodology thus provides an unbiased framework to identify key virulence factors in a bacterium that has not been previously recognized as a significant pathogen. On a more general note, this work provides yet another example of the conversion of a relatively benign organism, such as the reference strain, into a dangerous pathogen through phage infection and transfer of mobile genetic elements.

## MATERIALS AND METHODS

The Paenibacillus thiaminolyticus reference strain NRRL B-4156^T^ was obtained from the U.S. Department of Agriculture Agricultural Research Service Culture Collection (NRRL, Peoria, IL).

### Clinical microbiology.

Isolation and genome assembly of the Mbale strain were previously described (13). To recover additional clinical isolates, 1-mL aliquots of CSF from 63 PIH patients were inoculated as previously described (13). Two CSF samples were positive for growth in culture bottles. For specific details, see the supplemental material.

### Bacterial genome sequence and assembly.

Bacterial cultures were inoculated in either Luria-Bertani (LB) broth from monoisolates or anaerobic culture bottles. DNA was extracted using either the Prep Cell culture DNA isolation protocol (Bionano Genomics, San Diego, CA) or the Zymobiomics DNA kit following the manufacturer’s protocol as previously described (23). For specific details, see the supplemental material.

Genomes of strains Mbale and NRRL B-4156 were assembled as previously described (23). The genomes of two additional clinical isolates, Mbale2 and Mbale3, were assembled using the same protocol. Briefly, long reads generated on a MinION instrument (Oxford Nanopore) were preprocessed using Albacore, assembled with Canu (79), and corrected with Pilon (80).

### Genome annotation and protein comparison.

We used the Prokaryotic Genome Annotation Pipeline (PGAP) (28, 81) through RefSeq (28) and RASTtk (38) annotation, summarized by the PATRIC database (29), to annotate the three clinical isolates and reference strains. Genome annotation and a function-based comparison were performed using RASTtk (38) with the default parameters. The PATRIC database (29) was also used for protein comparisons of the annotations (70% minimal coverage and 30% minimal identity) and the proteomics (50% minimal coverage and 30% minimal identity). Mapping of proteins to the genomic assembly was done using CGView (82). OrthoVenn2 (83) with default settings was used to compare the predicted proteins with proteins from previously sequenced related bacteria, and gene ontology terms for the mapped genes were plotted with ggplot2 (84) in the R statistical computing program version 4.0.4. An average nucleotide identity calculator was used to compare the overall nucleotide identity (25). PilFind using default settings was used to identify the N-terminal pilin motifs in hypothetical proteins (42). PHASTER was used to identify specific phage insertions in each genome (24).

### RNA isolation, sequencing, and analysis.

Bacteria were grown in M9 mineral medium with 0.4% glucose or LB medium, and samples were prepared at five stages of growth from both cultures to evaluate the span of gene expression. RNA was isolated using the Direct-zol RNA miniprep kit (Zymo, USA) following the manufacturer’s protocol with bead beating and a DNase I treatment protocol. RNA was prepared for sequencing with the NEBNext rRNA depletion kit (E7850; New England Biolabs [NEB], USA), followed by TruSeq Stranded Total RNA Prep (kit from Illumina, USA) following the manufacturer’s protocol. Counts were generated with HTSeq aligned to the respective PGAP annotated genome. Comparisons were done with the Mbale and reference strains aligned to the Mbale annotation. Normalization and exploratory analyses were done with DESeq2 (85) using the R statistical programming hclust package, which utilizes the complete linkage method with Euclidean distances and is represented using the pheatmap package in R.

### Proteomic preparations and analysis of isolates.

Three liquid cultures from three separate colonies each of Mbale and the reference strains were analyzed concurrently. All samples were digested with trypsin after protein denaturation, reduction, and alkylation essentially as described previously (86, 87). Data from the mass spectrometer were converted to peak lists, and the MS2 spectra were analyzed using Peaks software (88). For specific details, see the supplemental material.

### Genome editing with CRISPR-Cas9.

All plasmids for genome editing were constructed in E. coli DH5α [*fhuA2 lac*Δ*U169 phoA glnV44* ϕ80*lacZ*ΔM15 *gyrA96 recA1 relA1 endA1 thi-1 hsdR17*] and, after sequencing confirmation, were transformed into E. coli BW29427 [*RP4-2*(*tetS kan1360*::FRT) *thrB1004* Δ*lacZ58*(M15) Δ*dapA1341*::(*erm pir*^+^) *rpsL*(*strR thi hsdS pro*)] (The Coli Genetic Stock Center) selecting on LB medium containing 50 μg/mL chloramphenicol and 100 μg/mL diaminopimelic acid (DAP). All plasmid constructions were performed with the appropriate enzymes from New England Biolabs.

For genome editing, a vector was derived from plasmid pJOE9734 (Bacillus Genetic Stock Center) (89, 90) modified with an inserted guide RNA and homologous directed region and then conjugated into *P. thiaminolyticus* Mbale for cas9 induction and genome editing. For specific details, see the supplemental material.

### Virulence testing using C57BL/6J mice.

All animal experiments were performed with oversight by the Penn State Institutional Animal Care and Use Committee, and with Institutional Biosafety Committee approval at biosafety level 2 (BSL2). The virulence of Mbale and Δ(*pilT pilC pilB*) strains was tested using the mouse infection model as previously described (13). For details, see the supplemental material.

### PCR of *pilT* gene.

DNA was isolated as described above from either CSF or blood from patients with hydrocephalus. PCR of the *pilT* gene was performed with OneTaq (NEB, M0207) with 500 nM forward (GATCATAATCAATGAGCCCGGTCATGG) and reverse (CTTGTGCGAAGGCGCTGCGA) primers in 25-μL reaction mixtures. Amplified products were separated on a 1% agarose gel and visualized after staining with ethidium bromide, with scoring for the presence of a 200-bp product from *pilT*.

### Data availability.

The genomes of the reference strains NRRL B-4156 (GenBank accession number CP041405) and strain Mbale (GenBank number JAALJM000000000) were described previously (23). The genomes of two additional clinical isolates, Mbale2 and Mbale3, were deposited in GenBank with accession numbers CP092990 and CP092991, respectively. The corresponding BioProject accession numbers are listed in Table 1.

## References

[1] Dewan MC, Rattani A, Mekary R, Glancz LJ, Yunusa I, Baticulon RE, Fieggen G, Wellons JC, III, Park KB, Warf BC. 2019. Global hydrocephalus epidemiology and incidence: systematic review and meta-analysis. J Neurosurg 130:1065–1079. doi:10.3171/2017.10.JNS17439. 29701543

[2] Isaacs AM, Riva-Cambrin J, Yavin D, Hockley A, Pringsheim TM, Jette N, Lethebe BC, Lowerison M, Dronyk J, Hamilton MG. 2018. Age-specific global epidemiology of hydrocephalus: systematic review, metanalysis and global birth surveillance. PLoS One 13:e0204926. doi:10.1371/journal.pone.0204926. 30273390 PMC6166961

[3] Warf BC. 2005. Hydrocephalus in Uganda: the predominance of infectious origin and primary management with endoscopic third ventriculostomy. J Neurosurg 102:1–15. doi:10.3171/ped.2005.102.1.0001. 16206728

[4] Warf BC, East African Neurosurgical Research Collaboration. 2010. Pediatric hydrocephalus in East Africa: prevalence, causes, treatments, and strategies for the future. World Neurosurg 73:296–300. doi:10.1016/j.wneu.2010.02.009. 20849782

[5] Kulkarni AV, Schiff SJ, Mbabazi-Kabachelor E, Mugamba J, Ssenyonga P, Donnelly R, Levenbach J, Monga V, Peterson M, MacDonald M, Cherukuri V, Warf BC. 2017. Endoscopic treatment versus shunting for infant hydrocephalus in Uganda. N Engl J Med 377:2456–2464. doi:10.1056/NEJMoa1707568. 29262276 PMC5784827

[6] Warf BC, Alkire BC, Bhai S, Hughes C, Schiff SJ, Vincent JR, Meara JG. 2011. Costs and benefits of neurosurgical intervention for infant hydrocephalus in sub-Saharan Africa. J Neurosurg Pediatr 8:509–521. doi:10.3171/2011.8.PEDS11163. 22044378

[7] Sinnar SA, Schiff SJ. 2020. The problem of microbial dark matter in neonatal sepsis. Emerg Infect Dis 26:2543–2548. doi:10.3201/eid2611.200004. 33080169 PMC7588532

[8] Karimy JK, Reeves BC, Damisah E, Duy PQ, Antwi P, David W, Wang K, Schiff SJ, Limbrick DD, Jr, Alper SL, Warf BC, Nedergaard M, Simard JM, Kahle KT. 2020. Inflammation in acquired hydrocephalus: pathogenic mechanisms and therapeutic targets. Nat Rev Neurol 16:285–296. doi:10.1038/s41582-020-0321-y. 32152460 PMC7375440

[9] Vinchon M, Rekate H, Kulkarni AV. 2012. Pediatric hydrocephalus outcomes: a review. Fluids Barriers CNS 9:18. doi:10.1186/2045-8118-9-18. 22925451 PMC3584674

[10] Kiwanuka J, Bazira J, Mwanga J, Tumusiime D, Nyesigire E, Lwanga N, Warf BC, Kapur V, Poss M, Schiff SJ. 2013. The microbial spectrum of neonatal sepsis in Uganda: recovery of culturable bacteria in mother-infant pairs. PLoS One 8:e72775. doi:10.1371/journal.pone.0072775. 24013829 PMC3754959

[11] Li L, Padhi A, Ranjeva SL, Donaldson SC, Warf BC, Mugamba J, Johnson D, Opio Z, Jayarao B, Kapur V, Poss M, Schiff SJ. 2011. Association of bacteria with hydrocephalus in Ugandan infants. J Neurosurg Pediatr 7:73–87. doi:10.3171/2010.9.PEDS10162. 21194290

[12] Schiff SJ, Kiwanuka J, Riggio G, Nguyen L, Mu K, Sproul E, Bazira J, Mwanga-Amumpaire J, Tumusiime D, Nyesigire E, Lwanga N, Bogale KT, Kapur V, Broach JR, Morton SU, Warf BC, Poss M. 2016. Separating putative pathogens from background contamination with principal orthogonal decomposition: evidence for Leptospira in the Ugandan neonatal septisome. Front Med (Lausanne) 3:22. doi:10.3389/fmed.2016.00022. 27379237 PMC4904006

[13] Paulson JN, Williams BL, Hehnly C, Mishra N, Sinnar SA, Zhang L, Ssentongo P, Mbabazi-Kabachelor E, Wijetunge DSS, von Bredow B, Mulondo R, Kiwanuka J, Bajunirwe F, Bazira J, Bebell LM, Burgoine K, Couto-Rodriguez M, Ericson JE, Erickson T, Ferrari M, Gladstone M, Guo C, Haran M, Hornig M, Isaacs AM, Kaaya BN, Kangere SM, Kulkarni AV, Kumbakumba E, Li X, Limbrick DD, Jr, Magombe J, Morton SU, Mugamba J, Ng J, Olupot-Olupot P, Onen J, Peterson MR, Roy F, Sheldon K, Townsend R, Weeks AD, Whalen AJ, Quackenbush J, Ssenyonga P, Galperin MY, Almeida M, Atkins H, Warf BC, Lipkin WI, et al. 2020. Paenibacillus infection with frequent viral coinfection contributes to postinfectious hydrocephalus in Ugandan infants. Sci Transl Med 12:eaba0565. doi:10.1126/scitranslmed.aba0565. 32998967 PMC7774825

[14] Grady EN, MacDonald J, Liu L, Richman A, Yuan ZC. 2016. Current knowledge and perspectives of Paenibacillus: a review. Microb Cell Fact 15:203. doi:10.1186/s12934-016-0603-7. 27905924 PMC5134293

[15] Genersch E. 2010. American foulbrood in honeybees and its causative agent, Paenibacillus larvae. J Invertebr Pathol 103(Suppl 1):S10–S19. doi:10.1016/j.jip.2009.06.015. 19909971

[16] DeLeon SD, Welliver RC, Sr. 2016. Paenibacillus alvei sepsis in a neonate. Pediatr Infect Dis J 35:358. doi:10.1097/INF.0000000000001003. 26866854

[17] Hunt B, Rogers C, Blais RM, Adachi K, Sathyavagiswaran L. 2021. Paenibacillus sepsis and meningitis in a premature infant: a case report. Am J Forensic Med Pathol 42:96–98. doi:10.1097/PAF.0000000000000610. 32852292

[18] Leao RS, Pereira RH, Ferreira AG, Lima AN, Albano RM, Marques EA. 2010. First report of Paenibacillus cineris from a patient with cystic fibrosis. Diagn Microbiol Infect Dis 66:101–103. doi:10.1016/j.diagmicrobio.2009.06.011. 19709843

[19] Ouyang J, Pei Z, Lutwick L, Dalal S, Yang L, Cassai N, Sandhu K, Hanna B, Wieczorek RL, Bluth M, Pincus MR. 2008. Case report: Paenibacillus thiaminolyticus: a new cause of human infection, inducing bacteremia in a patient on hemodialysis. Ann Clin Lab Sci 38:393–400. 18988935 PMC2955490

[20] Quenard F, Aubry C, Palmieri M, Edouard S, Parola P, Lagier JC. 2016. First case of bone infection caused by Paenibacillus turicensis. New Microbes New Infect 11:45–46. doi:10.1016/j.nmni.2016.02.004. 27257492 PMC4877404

[21] Saez-Nieto JA, Medina-Pascual MJ, Carrasco G, Garrido N, Fernandez-Torres MA, Villalon P, Valdezate S. 2017. Paenibacillus spp. isolated from human and environmental samples in Spain: detection of 11 new species. New Microbes New Infect 19:19–27. doi:10.1016/j.nmni.2017.05.006. 28702198 PMC5484988

[22] Morton SU, Hehnly C, Burgoine K, Ssentongo P, Ericson JE, Kumar MS, Hagmann C, Fronterre C, Smith J, Movassagh M, Streck N, Bebell L, Bazira J, Kumbakumba E, Kaaya BN, Natukwatsa D, Nalule E, Magombe J, Erickson T, Ngonzi J, Moses O, Olupot-Olupot P, Onen J, Ssenyonga P, Mugamba J, Warf BC, Kulkami A, Lane J, Whalen AJ, Zhang L, Sheldon K, Meier F, Kiwanuka J, Broach JR, Paulson J, Schiff SJ. 2022. Paenibacillus infection causes neonatal sepsis and subsequent postinfectious hydrocephalus in Ugandan infants. Preprints Lancet doi:10.2139/ssrn.4016548.

[23] Hehnly C, Zhang L, Paulson JN, Almeida M, von Bredow B, Wijetunge DSS, Galperin MY, Sheldon K, Schiff SJ, Broach JR. 2020. Complete genome sequences of the human pathogen Paenibacillus thiaminolyticus Mbale and type strain P thiaminolyticus NRRL B-4156. Microbiol Resour Announc 9:e00181-20. doi:10.1128/MRA.00181-20. 32273361 PMC7380522

[24] Arndt D, Grant JR, Marcu A, Sajed T, Pon A, Liang Y, Wishart DS. 2016. PHASTER: a better, faster version of the PHAST phage search tool. Nucleic Acids Res 44:W16–W21. doi:10.1093/nar/gkw387. 27141966 PMC4987931

[25] Goris J, Konstantinidis KT, Klappenbach JA, Coenye T, Vandamme P, Tiedje JM. 2007. DNA-DNA hybridization values and their relationship to whole-genome sequence similarities. Int J Syst Evol Microbiol 57:81–91. doi:10.1099/ijs.0.64483-0. 17220447

[26] Rodriguez RL, Gunturu S, Harvey WT, Rossello-Mora R, Tiedje JM, Cole JR, Konstantinidis KT. 2018. The Microbial Genomes Atlas (MiGA) webserver: taxonomic and gene diversity analysis of Archaea and Bacteria at the whole genome level. Nucleic Acids Res 46:W282–W288. doi:10.1093/nar/gky467. 29905870 PMC6031002

[27] Darling AC, Mau B, Blattner FR, Perna NT. 2004. Mauve: multiple alignment of conserved genomic sequence with rearrangements. Genome Res 14:1394–1403. doi:10.1101/gr.2289704. 15231754 PMC442156

[28] Li W, O'Neill KR, Haft DH, DiCuccio M, Chetvernin V, Badretdin A, Coulouris G, Chitsaz F, Derbyshire MK, Durkin AS, Gonzales NR, Gwadz M, Lanczycki CJ, Song JS, Thanki N, Wang J, Yamashita RA, Yang M, Zheng C, Marchler-Bauer A, Thibaud-Nissen F. 2021. RefSeq: expanding the Prokaryotic Genome Annotation Pipeline reach with protein family model curation. Nucleic Acids Res 49:D1020–D1028. doi:10.1093/nar/gkaa1105. 33270901 PMC7779008

[29] Davis JJ, Wattam AR, Aziz RK, Brettin T, Butler R, Butler RM, Chlenski P, Conrad N, Dickerman A, Dietrich EM, Gabbard JL, Gerdes S, Guard A, Kenyon RW, Machi D, Mao C, Murphy-Olson D, Nguyen M, Nordberg EK, Olsen GJ, Olson RD, Overbeek JC, Overbeek R, Parrello B, Pusch GD, Shukla M, Thomas C, VanOeffelen M, Vonstein V, Warren AS, Xia F, Xie D, Yoo H, Stevens R. 2020. The PATRIC Bioinformatics Resource Center: expanding data and analysis capabilities. Nucleic Acids Res 48:D606–D612. doi:10.1093/nar/gkz943. 31667520 PMC7145515

[30] Rice K, Batul K, Whiteside J, Kelso J, Papinski M, Schmidt E, Pratasouskaya A, Wang D, Sullivan R, Bartlett C, Weadge JT, Van der Kamp MW, Moreno-Hagelsieb G, Suits MD, Horsman GP. 2019. The predominance of nucleotidyl activation in bacterial phosphonate biosynthesis. Nat Commun 10:3698. doi:10.1038/s41467-019-11627-6. 31420548 PMC6697681

[31] Hensbergen PJ, de Ru AH, Friggen AH, Corver J, Smits WK, van Veelen PA. 2022. New insights into the type A glycan modification of *Clostridioides difficile* flagellar protein flagellin C by phosphoproteomics analysis. J Biol Chem 298:101622. doi:10.1016/j.jbc.2022.101622. 35065968 PMC8861647

[32] Schneider K, Kastner CN, Meyer M, Wessel M, Dimroth P, Bott M. 2002. Identification of a gene cluster in *Klebsiella pneumoniae* which includes *citX*, a gene required for biosynthesis of the citrate lyase prosthetic group. J Bacteriol 184:2439–2446. doi:10.1128/JB.184.9.2439-2446.2002. 11948157 PMC134981

[33] Hu J, Jin K, He ZG, Zhang H. 2020. Citrate lyase CitE in *Mycobacterium tuberculosis* contributes to mycobacterial survival under hypoxic conditions. PLoS One 15:e0230786. doi:10.1371/journal.pone.0230786. 32302313 PMC7164622

[34] Martino GP, Perez CE, Magni C, Blancato VS. 2018. Implications of the expression of *Enterococcus faecalis* citrate fermentation genes during infection. PLoS One 13:e0205787. doi:10.1371/journal.pone.0205787. 30335810 PMC6193673

[35] Hsiao WW, Ung K, Aeschliman D, Bryan J, Finlay BB, Brinkman FS. 2005. Evidence of a large novel gene pool associated with prokaryotic genomic islands. PLoS Genet 1:e62. doi:10.1371/journal.pgen.0010062. 16299586 PMC1285063

[36] Perna NT, Plunkett G, III, Burland V, Mau B, Glasner JD, Rose DJ, Mayhew GF, Evans PS, Gregor J, Kirkpatrick HA, Posfai G, Hackett J, Klink S, Boutin A, Shao Y, Miller L, Grotbeck EJ, Davis NW, Lim A, Dimalanta ET, Potamousis KD, Apodaca J, Anantharaman TS, Lin J, Yen G, Schwartz DC, Welch RA, Blattner FR. 2001. Genome sequence of enterohaemorrhagic Escherichia coli O157:H7. Nature 409:529–533. doi:10.1038/35054089. 11206551

[37] Durrant MG, Li MM, Siranosian BA, Montgomery SB, Bhatt AS. 2020. A ioinformatic analysis of integrative mobile genetic elements highlights their role in bacterial adaptation. Cell Host Microbe 27:140–153.E9. doi:10.1016/j.chom.2019.10.022. 31862382 PMC6952549

[38] Brettin T, Davis JJ, Disz T, Edwards RA, Gerdes S, Olsen GJ, Olson R, Overbeek R, Parrello B, Pusch GD, Shukla M, Thomason JA, III, Stevens R, Vonstein V, Wattam AR, Xia F. 2015. RASTtk: a modular and extensible implementation of the RAST algorithm for building custom annotation pipelines and annotating batches of genomes. Sci Rep 5:8365. doi:10.1038/srep08365. 25666585 PMC4322359

[39] Szaniawski MA, Spivak AM. 2019. Recurrent Paenibacillus infection. Oxf Med Case Rep 2019:omz034. 10.1093/omcr/omz034 PMC6544421 31198570

[40] Szklarczyk D, Gable AL, Nastou KC, Lyon D, Kirsch R, Pyysalo S, Doncheva NT, Legeay M, Fang T, Bork P, Jensen LJ, von Mering C. 2021. The STRING database in 2021: customizable protein-protein networks, and functional characterization of user-uploaded gene/measurement sets. Nucleic Acids Res 49:D605–D612. doi:10.1093/nar/gkaa1074. 33237311 PMC7779004

[41] Melville S, Craig L. 2013. Type IV pili in Gram-positive bacteria. Microbiol Mol Biol Rev 77:323–341. doi:10.1128/MMBR.00063-12. 24006467 PMC3811610

[42] Imam S, Chen Z, Roos DS, Pohlschroder M. 2011. Identification of surprisingly diverse type IV pili, across a broad range of gram-positive bacteria. PLoS One 6:e28919. doi:10.1371/journal.pone.0028919. 22216142 PMC3244431

[43] Gurung I, Spielman I, Davies MR, Lala R, Gaustad P, Biais N, Pelicic V. 2016. Functional analysis of an unusual type IV pilus in the Gram-positive Streptococcus sanguinis. Mol Microbiol 99:380–392. doi:10.1111/mmi.13237. 26435398 PMC4832360

[44] Roelofs KG, Jones CJ, Helman SR, Shang X, Orr MW, Goodson JR, Galperin MY, Yildiz FH, Lee VT. 2015. Systematic identification of cyclic-di-GMP binding proteins in *Vibrio cholerae* reveals a novel class of cyclic-di-GMP-binding ATPases associated with type II secretion systems. PLoS Pathog 11:e1005232. doi:10.1371/journal.ppat.1005232. 26506097 PMC4624772

[45] Wang YC, Chin KH, Tu ZL, He J, Jones CJ, Sanchez DZ, Yildiz FH, Galperin MY, Chou SH. 2016. Nucleotide binding by the widespread high-affinity cyclic di-GMP receptor MshEN domain. Nat Commun 7:12481. doi:10.1038/ncomms12481. 27578558 PMC5013675

[46] Hendrick WA, Orr MW, Murray SR, Lee VT, Melville SB. 2017. Cyclic Di-GMP binding by an assembly ATPase (PilB2) and control of type IV pilin polymerization in the Gram-positive pathogen Clostridium perfringens. J Bacteriol 199:e00034-17. doi:10.1128/JB.00034-17. 28242722 PMC5405213

[47] McKee RW, Aleksanyan N, Garrett EM, Tamayo R. 2018. Type IV pili promote Clostridium difficile adherence and persistence in a mouse model of infection. Infect Immun 86:e00943-17. doi:10.1128/IAI.00943-17. 29483294 PMC5913833

[48] Ash C, Priest FG, Collins MD. 1993. Molecular identification of rRNA group 3 bacilli (Ash, Farrow, Wallbanks and Collins) using a PCR probe test. Proposal for the creation of a new genus Paenibacillus. Antonie Van Leeuwenhoek 64:253–260. doi:10.1007/BF00873085. 8085788

[49] Gallegos MT, Schleif R, Bairoch A, Hofmann K, Ramos JL. 1997. Arac/XylS family of transcriptional regulators. Microbiol Mol Biol Rev 61:393–410. doi:10.1128/mmbr.61.4.393-410.1997. 9409145 PMC232617

[50] Martin RG, Rosner JL. 2001. The AraC transcriptional activators. Curr Opin Microbiol 4:132–137. doi:10.1016/s1369-5274(00)00178-8. 11282467

[51] Saadat A, Melville SB. 2021. Holin-dependent secretion of the large clostridial toxin TpeL by Clostridium perfringens. J Bacteriol 203:e00580-20. doi:10.1128/JB.00580-20. 33526612 PMC8088506

[52] Kovacs-Simon A, Titball RW, Michell SL. 2011. Lipoproteins of bacterial pathogens. Infect Immun 79:548–561. doi:10.1128/IAI.00682-10. 20974828 PMC3028857

[53] Kurokawa K, Ryu KH, Ichikawa R, Masuda A, Kim MS, Lee H, Chae JH, Shimizu T, Saitoh T, Kuwano K, Akira S, Dohmae N, Nakayama H, Lee BL. 2012. Novel bacterial lipoprotein structures conserved in low-GC content gram-positive bacteria are recognized by Toll-like receptor 2. J Biol Chem 287:13170–13181. doi:10.1074/jbc.M111.292235. 22303020 PMC3339964

[54] Isaacs AM, Morton SU, Movassagh M, Zhang Q, Hehnly C, Zhang L, Morales DM, Sinnar SA, Ericson JE, Mbabazi-Kabachelor E, Ssenyonga P, Onen J, Mulondo R, Hornig M, Warf BC, Broach JR, Townsend RR, Limbrick DD, Jr, Paulson JN, Schiff SJ. 2021. Immune activation during Paenibacillus brain infection in African infants with frequent cytomegalovirus co-infection. iScience 24:102351. doi:10.1016/j.isci.2021.102351. 33912816 PMC8065213

[55] Fuchs A, Bielicki J, Mathur S, Sharland M, Van Den Anker JN. 2018. Reviewing the WHO guidelines for antibiotic use for sepsis in neonates and children. Paediatr Int Child Health 38:S3–S15. doi:10.1080/20469047.2017.1408738. 29790842 PMC6176768

[56] Burrows LL. 2005. Weapons of mass retraction. Mol Microbiol 57:878–888. doi:10.1111/j.1365-2958.2005.04703.x. 16091031

[57] Giltner CL, Nguyen Y, Burrows LL. 2012. Type IV pilin proteins: versatile molecular modules. Microbiol Mol Biol Rev 76:740–772. doi:10.1128/MMBR.00035-12. 23204365 PMC3510520

[58] Pelicic V. 2008. Type IV pili: e pluribus unum? Mol Microbiol 68:827–837. doi:10.1111/j.1365-2958.2008.06197.x. 18399938

[59] Muschiol S, Aschtgen MS, Nannapaneni P, Henriques-Normark B. 2019. Gram-positive type IV pili and competence. Microbiol Spectr 7:PSIB-0011-2018. doi:10.1128/microbiolspec.PSIB-0011-2018. PMC11588153 30737914

[60] Pelicic V. 2019. Monoderm bacteria: the new frontier for type IV pilus biology. Mol Microbiol 112:1674–1683. doi:10.1111/mmi.14397. 31556183 PMC6916266

[61] Rozman V, Accetto T, Duncan SH, Flint HJ, Vodovnik M. 2021. Type IV pili are widespread among non-pathogenic Gram-positive gut bacteria with diverse carbohydrate utilization patterns. Environ Microbiol 23:1527–1540. doi:10.1111/1462-2920.15362. 33331146

[62] Ayers M, Howell PL, Burrows LL. 2010. Architecture of the type II secretion and type IV pilus machineries. Future Microbiol 5:1203–1218. doi:10.2217/fmb.10.76. 20722599

[63] Chang YW, Rettberg LA, Treuner-Lange A, Iwasa J, Sogaard-Andersen L, Jensen GJ. 2016. Architecture of the type IVa pilus machine. Science 351:aad2001. doi:10.1126/science.aad2001. 26965631 PMC5929464

[64] Purcell EB, Tamayo R. 2016. Cyclic diguanylate signaling in Gram-positive bacteria. FEMS Microbiol Rev 40:753–773. doi:10.1093/femsre/fuw013. 27354347 PMC5007281

[65] Chlebek JL, Denise R, Craig L, Dalia AB. 2021. Motor-independent retraction of type IV pili is governed by an inherent property of the pilus filament. Proc Natl Acad Sci USA 118:e2102780118. doi:10.1073/pnas.2102780118. 34789573 PMC8617508

[66] Siefert JL, Larios-Sanz M, Nakamura LK, Slepecky RA, Paul JH, Moore ER, Fox GE, Jurtshuk P, Jr. 2000. Phylogeny of marine Bacillus isolates from the Gulf of Mexico. Curr Microbiol 41:84–88. doi:10.1007/s002840010098. 10856371

[67] Massilamany C, Mohammed A, Loy JD, Purvis T, Krishnan B, Basavalingappa RH, Kelley CM, Guda C, Barletta RG, Moriyama EN, Smith TP, Reddy J. 2016. Whole genomic sequence analysis of Bacillus infantis: defining the genetic blueprint of strain NRRL B-14911, an emerging cardiopathogenic microbe. BMC Genomics 17(Suppl 7):511. doi:10.1186/s12864-016-2900-2. 27557119 PMC5001198

[68] Ko KS, Oh WS, Lee MY, Lee JH, Lee H, Peck KR, Lee NY, Song JH. 2006. Bacillus infantis sp. nov. and Bacillus idriensis sp. nov., isolated from a patient with neonatal sepsis. Int J Syst Evol Microbiol 56:2541–2544. doi:10.1099/ijs.0.64213-0. 17082387

[69] Bordeleau E, Purcell EB, Lafontaine DA, Fortier LC, Tamayo R, Burrus V. 2015. Cyclic di-GMP riboswitch-regulated type IV pili contribute to aggregation of *Clostridium difficile*. J Bacteriol 197:819–832. doi:10.1128/JB.02340-14. 25512308 PMC4325102

[70] Maldarelli GA, Piepenbrink KH, Scott AJ, Freiberg JA, Song Y, Achermann Y, Ernst RK, Shirtliff ME, Sundberg EJ, Donnenberg MS, von Rosenvinge EC. 2016. Type IV pili promote early biofilm formation by *Clostridium difficile*. Pathog Dis 74:ftw061. doi:10.1093/femspd/ftw061. 27369898 PMC5985507

[71] Soncini SR, Hartman AH, Gallagher TM, Camper GJ, Jensen RV, Melville SB. 2020. Changes in the expression of genes encoding type IV pili-associated proteins are seen when Clostridium perfringens is grown in liquid or on surfaces. BMC Genomics 21:45. doi:10.1186/s12864-020-6453-z. 31937237 PMC6958937

[72] Barnier JP, Euphrasie D, Join-Lambert O, Audry M, Schonherr-Hellec S, Schmitt T, Bourdoulous S, Coureuil M, Nassif X, El Behi M. 2021. Type IV pilus retraction enables sustained bacteremia and plays a key role in the outcome of meningococcal sepsis in a humanized mouse model. PLoS Pathog 17:e1009299. doi:10.1371/journal.ppat.1009299. 33592056 PMC7909687

[73] Denis K, Le Bris M, Le Guennec L, Barnier JP, Faure C, Gouge A, Bouzinba-Segard H, Jamet A, Euphrasie D, Durel B, Barois N, Pelissier P, Morand PC, Coureuil M, Lafont F, Join-Lambert O, Nassif X, Bourdoulous S. 2019. Targeting type IV pili as an antivirulence strategy against invasive meningococcal disease. Nat Microbiol 4:972–984. doi:10.1038/s41564-019-0395-8. 30911127

[74] Herrington DA, Hall RH, Losonsky G, Mekalanos JJ, Taylor RK, Levine MM. 1988. Toxin, toxin-coregulated pili, and the toxR regulon are essential for Vibrio cholerae pathogenesis in humans. J Exp Med 168:1487–1492. doi:10.1084/jem.168.4.1487. 2902187 PMC2189073

[75] Kirn TJ, Bose N, Taylor RK. 2003. Secretion of a soluble colonization factor by the TCP type 4 pilus biogenesis pathway in Vibrio cholerae. Mol Microbiol 49:81–92. doi:10.1046/j.1365-2958.2003.03546.x. 12823812

[76] Bremm KD, Konig W, Pfeiffer P, Rauschen I, Theobald K, Thelestam M, Alouf JE. 1985. Effect of thiol-activated toxins (streptolysin O, alveolysin, and theta toxin) on the generation of leukotrienes and leukotriene-inducing and -metabolizing enzymes from human polymorphonuclear granulocytes. Infect Immun 50:844–851. doi:10.1128/iai.50.3.844-851.1985. 2866160 PMC261157

[77] Geoffroy C, Mengaud J, Alouf JE, Cossart P. 1990. Alveolysin, the thiol-activated toxin of Bacillus alvei, is homologous to listeriolysin O, perfringolysin O, pneumolysin, and streptolysin O and contains a single cysteine. J Bacteriol 172:7301–7305. doi:10.1128/jb.172.12.7301-7305.1990. 2254290 PMC210863

[78] Thelestam M, Alouf JE, Geoffroy C, Mollby R. 1981. Membrane-damaging action of alveolysin from Bacillus alvei. Infect Immun 32:1187–1192. doi:10.1128/iai.32.3.1187-1192.1981. 6894743 PMC351577

[79] Koren S, Walenz BP, Berlin K, Miller JR, Bergman NH, Phillippy AM. 2017. Canu: scalable and accurate long-read assembly via adaptive k-mer weighting and repeat separation. Genome Res 27:722–736. doi:10.1101/gr.215087.116. 28298431 PMC5411767

[80] Walker BJ, Abeel T, Shea T, Priest M, Abouelliel A, Sakthikumar S, Cuomo CA, Zeng Q, Wortman J, Young SK, Earl AM. 2014. Pilon: an integrated tool for comprehensive microbial variant detection and genome assembly improvement. PLoS One 9:e112963. doi:10.1371/journal.pone.0112963. 25409509 PMC4237348

[81] Tatusova T, DiCuccio M, Badretdin A, Chetvernin V, Nawrocki EP, Zaslavsky L, Lomsadze A, Pruitt KD, Borodovsky M, Ostell J. 2016. NCBI prokaryotic genome annotation pipeline. Nucleic Acids Res 44:6614–6624. doi:10.1093/nar/gkw569. 27342282 PMC5001611

[82] Stothard P, Grant JR, Van Domselaar G. 2019. Visualizing and comparing circular genomes using the CGView family of tools. Brief Bioinform 20:1576–1582. doi:10.1093/bib/bbx081. 28968859 PMC6781573

[83] Xu L, Dong Z, Fang L, Luo Y, Wei Z, Guo H, Zhang G, Gu YQ, Coleman-Derr D, Xia Q, Wang Y. 2019. OrthoVenn2: a web server for whole-genome comparison and annotation of orthologous clusters across multiple species. Nucleic Acids Res 47:W52–W58. doi:10.1093/nar/gkz333. 31053848 PMC6602458

[84] Wickham H. 2009. ggplot2: elegant graphics for data analysis. Springer, New York, NY.

[85] Love MI, Huber W, Anders S. 2014. Moderated estimation of fold change and dispersion for RNA-seq data with DESeq2. Genome Biol 15:550. doi:10.1186/s13059-014-0550-8. 25516281 PMC4302049

[86] Chen ZW, Fuchs K, Sieghart W, Townsend RR, Evers AS. 2012. Deep amino acid sequencing of native brain GABAA receptors using high-resolution mass spectrometry. Mol Cell Proteomics 11:M111-011445. doi:10.1074/mcp.M111.011445. PMC3270104 22338125

[87] Wiśniewski JR, Zougman A, Nagaraj N, Mann M. 2009. Universal sample preparation method for proteome analysis. Nat Methods 6:359–362. doi:10.1038/nmeth.1322. 19377485

[88] Ma B, Zhang K, Hendrie C, Liang C, Li M, Doherty-Kirby A, Lajoie G. 2003. PEAKS: powerful software for peptide de novo sequencing by tandem mass spectrometry. Rapid Commun Mass Spectrom 17:2337–2342. doi:10.1002/rcm.1196. 14558135

[89] Toymentseva AA, Altenbuchner J. 2019. New CRISPR-Cas9 vectors for genetic modifications of Bacillus species. FEMS Microbiol Lett 366:fny284. doi:10.1093/femsle/fny284. 30520985

[90] Altenbuchner J. 2016. Editing of the Bacillus subtilis genome by the CRISPR-Cas9 sSystem. Appl Environ Microbiol 82:5421–5427. doi:10.1128/AEM.01453-16. 27342565 PMC4988203

[91] Düvel J, Bense S, Möller S, Bertinetti D, Schwede F, Morr M, Eckweiler D, Genieser HG, Jänsch L, Herberg FW, Frank R, Häussler S. 2016. Application of synthetic peptide arrays to uncover cyclic di-GMP binding motifs. J Bacteriol 198:138–146. doi:10.1128/JB.00377-15. 26324453 PMC4686192

[92] Bischof TS, Hahn BL, Sohnle PG. 2007. Characteristics of spore germination in a mouse model of cutaneous anthrax. J Infect Dis 195:888–894. doi:10.1086/511824. 17299720

[93] Junkermeier EH, Hengge R. 2021. A novel locally c-di-GMP-controlled exopolysaccharide synthase required for bacteriophage N4 infection of *Escherichia coli*. mBio 12:e03249-21. doi:10.1128/mbio.03249-21. 34903052 PMC8669469

[94] Sellner B, Prakapaitė R, van Berkum M, Heinemann M, Harms A, Jenal U. 2021. A new sugar for an old phage: a c-di-GMP-dependent polysaccharide pathway sensitizes *Escherichia coli* for bacteriophage infection. mBio 12:e03246-21. doi:10.1128/mbio.03246-21. 34903045 PMC8669472

[95] Blum M, Chang HY, Chuguransky S, Grego T, Kandasaamy S, Mitchell A, Nuka G, Paysan-Lafosse T, Qureshi M, Raj S, Richardson L, Salazar GA, Williams L, Bork P, Bridge A, Gough J, Haft DH, Letunic I, Marchler-Bauer A, Mi H, Natale DA, Necci M, Orengo CA, Pandurangan AP, Rivoire C, Sigrist CJA, Sillitoe I, Thanki N, Thomas PD, Tosatto SCE, Wu CH, Bateman A, Finn RD. 2021. The InterPro protein families and domains database: 20 years on. Nucleic Acids Res 49:D344–D354. doi:10.1093/nar/gkaa977. 33156333 PMC7778928

[96] Galperin MY, Wolf YI, Makarova KS, Vera Alvarez R, Landsman D, Koonin EV. 2021. COG database update: focus on microbial diversity, model organisms, and widespread pathogens. Nucleic Acids Res 49:D274–D281. doi:10.1093/nar/gkaa1018. 33167031 PMC7778934

[97] Mistry J, Chuguransky S, Williams L, Qureshi M, Salazar GA, Sonnhammer ELL, Tosatto SCE, Paladin L, Raj S, Richardson LJ, Finn RD, Bateman A. 2021. Pfam: the protein families database in 2021. Nucleic Acids Res 49:D412–D419. doi:10.1093/nar/gkaa913. 33125078 PMC7779014

[98] Chernyatina AA, Low HH. 2019. Core architecture of a bacterial type II secretion system. Nat Commun 10:5437. doi:10.1038/s41467-019-13301-3. 31780649 PMC6882859

[99] Filloux A. 2004. The underlying mechanisms of type II protein secretion. Biochim Biophys Acta 1694:163–179. doi:10.1016/j.bbamcr.2004.05.003. 15546665

[100] Pugsley AP, Francetic O, Possot OM, Sauvonnet N, Hardie KR. 1997. Recent progress and future directions in studies of the main terminal branch of the general secretory pathway in Gram-negative bacteria–a review. Gene 192:13–19. doi:10.1016/s0378-1119(96)00803-7. 9224869

[101] Burrows LL. 2012. *Pseudomonas aeruginosa* twitching motility: type IV pili in action. Annu Rev Microbiol 66:493–520. doi:10.1146/annurev-micro-092611-150055. 22746331

[102] Varga JJ, Nguyen V, O'Brien DK, Rodgers K, Walker RA, Melville SB. 2006. Type IV pili-dependent gliding motility in the Gram-positive pathogen *Clostridium perfringens* and other Clostridia. Mol Microbiol 62:680–694. doi:10.1111/j.1365-2958.2006.05414.x. 16999833

