# Supplementary material for: Type IV Pili Are a Critical Virulence Factor in Clinical Isolates of Paenibacillus thiaminolyticus
Source: mBio. 2022 Nov 14;13(6):e02688-22. doi: 10.1128/mbio.02688-22 (PMC9765702; doi:10.1128/mbio.02688-22)
Supplement: FIG S1 [file mbio.02688-22-s0001.docx]

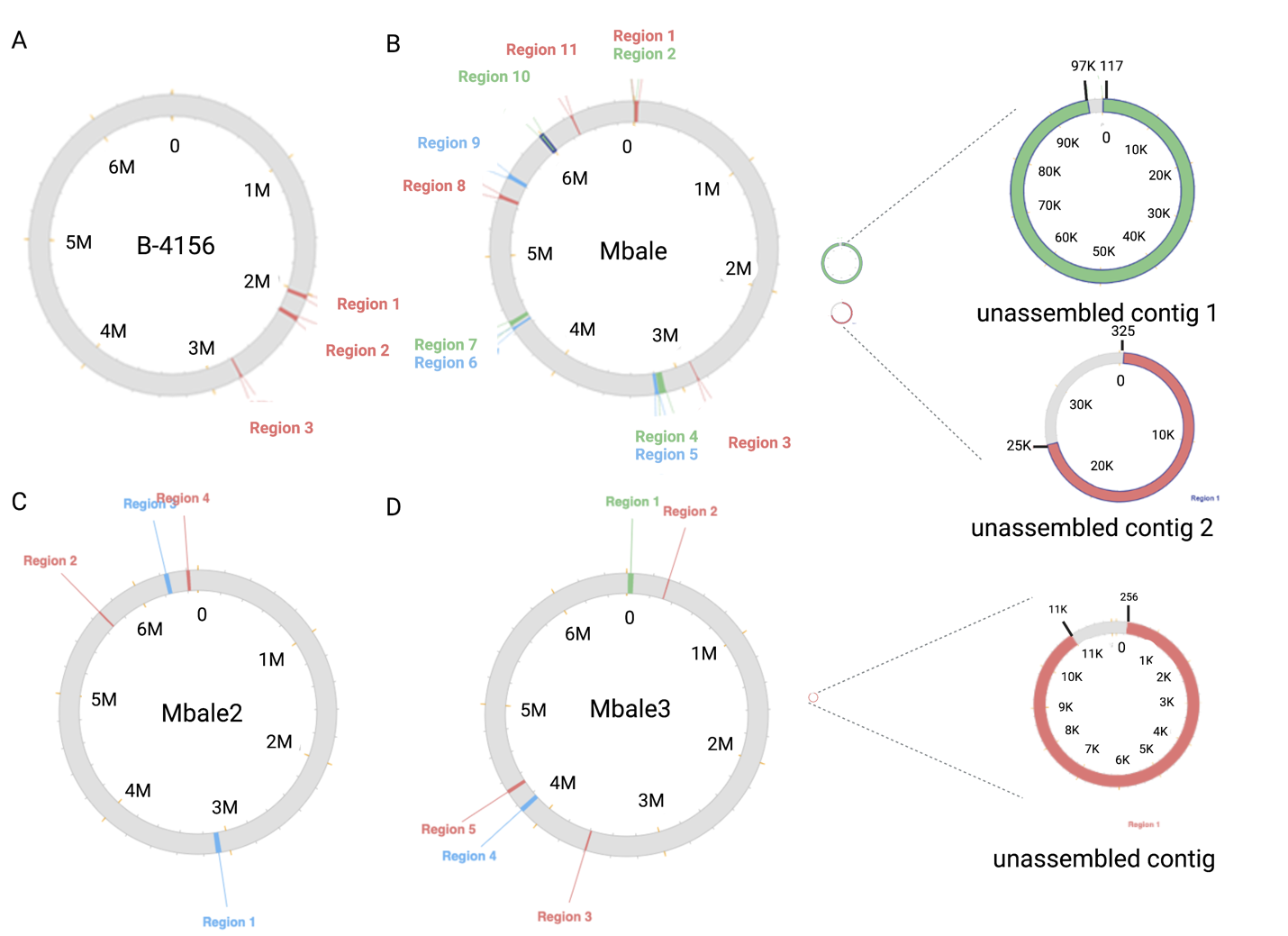


Fig S1. **Phage regions identified in each clinical isolate**. The results from PHASTER for strains A) B-4156, B) Mbale, C) Mbale2, and D) Mbale3. The colors correspond to the score that PHASTER assigns to the phage identification region, indicating an intact phage (green), questionable (blue) and incomplete (red). This score reflects the extent of presence of appropriate proteins for a functional phage.
