# Supplementary material for: Type IV Pili Are a Critical Virulence Factor in Clinical Isolates of Paenibacillus thiaminolyticus
Source: mBio. 2022 Nov 14;13(6):e02688-22. doi: 10.1128/mbio.02688-22 (PMC9765702; doi:10.1128/mbio.02688-22)
Supplement: TABLE S1 [file mbio.02688-22-s0006.docx]

Table S1. **Results from biochemical testing of all four isolates**.

| Test | Carbohydrate | B-4156 | | Mbale | | Mbale2 | | Mbale3 | |
| --- | --- | --- | --- | --- | --- | --- | --- | --- | --- |
|  |  | 24 hr | 48 hr | 24 hr | 48 hr | 24 hr | 48 hr | 24 hr | 48 hr |
| Control (NG = no growth) | | NG | NG | NG | NG | NG | NG | NG | NG |
| GLY | glycerol | + | + | + | + | + | + | + | + |
| ERY | erythritol | - | - | - | - | - | - | - | - |
| DARA | D-arabinose | - | ? | - | ? | - | ? | - | ? |
| LARA | L-arabinose | - | - | - | - | - | - | - | - |
| RIB | D-ribose | + | + | + | + | + | + | + | + |
| DXYL | D-xylose | - | - | - | - | - | - | - | - |
| LXYL | L-xylose | - | - | - | - | - | - | - | - |
| ADO | D-adonitol | - | - | - | - | - | - | - | - |
| MDX | methyl-βD-xylopyranoside | - | - | - | - | - | - | - | - |
| GAL | D-galactose | ?/- | + | + | + | - | ? | - | + |
| GLU | D-glucose | + | + | + | + | + | + | + | + |
| FRU | D-fructose | + | + | + | + | + | + | + | + |
| MNE | D-mannose | + | + | + | + | V | + | + | + |
| SBE | L-sorbose | - | - | - | - | - | - | - | - |
| RHA | L-rhamnose | - | - | - | - | - | - | - | - |
| DUL | dulcitol | - | - | - | - | - | - | - | - |
| INO | inositol | - | ? | -/? | - | - | -/? | - | ? |
| MAN | D-manitol | - | - | - | - | - | - | - | - |
| SOR | D-sorbitol | - | - | - | - | - | - | - | - |
| MDM | methyl αD- annopyranoside | -/? | + | -/? | + | - | ? | - | ? |
| MDG | methyl αD- glucopyranoside | + | + | + | + | V | + | V | + |
| NAG | N-acetylglucosamine | + | + | + | + | + | + | + | + |
| AMY | amygdaline | + | + | + | + | + | + | V | + |
|  |  |  |  |  |  |  |  |  |  |
| ARB | arbutine | + | + | + | + | + | + | V | + |
| ESC | esculin | + | + | + | + | + | + | + | + |
| SAL | salicin | + | + | + | + | + | + | V | + |
| CEL | D-cellobiose | + | + | + | + | -/? | + | - | ? |
| MAL | D-maltose | + | + | + | + | V | + | V | + |
| LAC | D-lactose (origin bovine) | V | + | + | + | - | +/? | - | -/? |
| MEL | D-melobiose | + | + | + | + | V | + | - | + |
| SAC | D-saccharose | + | + | + | + | V | + | -/? | + |
| TRE | D-trehalose | + | + | + | + | + | + | + | + |
| INU | inulin | - | - | - | -/? | - | - | - | - |
| MLZ | D-melezitose | + | + | + | + | - | ? | - | - |
| RAF | D-rafinose | + | + | + | + | V | + | V | + |
| AMD | amidon | + | + | + | + | V | + | -/? | + |
| GLYG | glycogene | + | + | V | + | - | -/? | - | - |
| XLT | xylitol | - | - | - | -/? | - | - | - | - |
| GEN | gentiobiose | + | + | + | + | V | + | V | + |
| TUR | D-turanose | + | + | + | + | V | + | - | +/? |
| LYX | D-lyxose | - | - | - | - | - | - | - | - |
| TAG | D-tagatose | - | - | - | - | - | - | - | - |
| DFUC | D-fucose | - | - | - | - | - | - | - | - |
| LFUC | L-fucose | -/? | + | -/? | +/? | - | ? | - | +/? |
| DARL | D-arabitol | - | - | - | - | - | - | - | - |
| LARL | L-arabitol | - | - | - | - | - | - | - | - |
| GNT | potassium gluconate | -/? | + | -/? | ? | - | +/? | - | +/? |
| 2KG | potassium 2-cetogluconate | - | - | - | - | - | - | - | - |
| 5KG | potassium 5-cetogluconate | - | -/? | - | - | - | - | - | - |
| Paenibacillus thiaminolyticus identification (%) | | 76-99.9 | 99.9 | 99.6-99.9 | 99.9 | ** | 97-98.6 | 89-95 | 91.6-98.9 |

**inconsistent organism ID

Abbreviations: +, positive; -, negative; ?, doubtful; V, variable across replicates
