# Supplementary material for: Type IV Pili Are a Critical Virulence Factor in Clinical Isolates of Paenibacillus thiaminolyticus
Source: mBio. 2022 Nov 14;13(6):e02688-22. doi: 10.1128/mbio.02688-22 (PMC9765702; doi:10.1128/mbio.02688-22)
Supplement: TABLE S2 [file mbio.02688-22-s0007.docx]

Table S2. **Gene ontology terms associated with 342 unique orthologous clusters in clinical isolates**.

| **Term** | **Genes** |
| --- | --- |
| GO:0043565 sequence-specific DNA binding | 16 |
| GO:0005886 plasma membrane | 12 |
| GO:0030435 sporulation resulting in formation of a cellular spore | 10 |
| GO:0003677 DNA binding | 9 |
| GO:0055085 transmembrane transport | 9 |
| GO:0003700 DNA-binding transcription factor activity | 6 |
| GO:0005524 ATP binding | 6 |
| GO:0008168 methyltransferase activity | 6 |
| GO:0016491 oxidoreductase activity | 6 |
| GO:0032923 organic phosphonate biosynthetic process | 6 |
| GO:0046677 response to antibiotic | 6 |
| GO:0032196 transposition | 4 |
| GO:0000155 phosphorelay sensor kinase activity | 3 |
| GO:0000162 tryptophan biosynthetic process | 3 |
| GO:0000271 polysaccharide biosynthetic process | 3 |
| GO:0006096 glycolytic process | 3 |
| GO:0006271 DNA strand elongation involved in DNA replication | 3 |
| GO:0006313 transposition, DNA-mediated | 3 |
| GO:0006364 rRNA processing | 3 |
| GO:0006629 lipid metabolic process | 3 |
| GO:0008270 zinc ion binding | 3 |
| GO:0009089 lysine biosynthetic process via diaminopimelate | 3 |
| GO:0009306 protein secretion | 3 |
| GO:0009307 DNA restriction-modification system | 3 |
| GO:0009636 response to toxic substance | 3 |
| GO:0015595 spermidine-importing ATPase activity | 3 |
| GO:0015628 protein secretion by the type II secretion system | 3 |
| GO:0016139 glycoside catabolic process | 3 |
| GO:0016301 kinase activity | 3 |
| GO:0016614 oxidoreductase activity, acting on CH-OH group of donors | 3 |
| GO:0016705 oxidoreductase activity, acting on paired donors, with incorporation or reduction of molecular oxygen | 3 |
| GO:0019350 teichoic acid biosynthetic process | 3 |
| GO:0019439 aromatic compound catabolic process | 3 |
| GO:0019509 L-methionine salvage from methylthioadenosine | 3 |
| GO:0019628 urate catabolic process | 3 |
| GO:0019700 organic phosphonate catabolic process | 3 |
| GO:0022857 transmembrane transporter activity | 3 |
