# Supplementary material for: Type IV Pili Are a Critical Virulence Factor in Clinical Isolates of Paenibacillus thiaminolyticus
Source: mBio. 2022 Nov 14;13(6):e02688-22. doi: 10.1128/mbio.02688-22 (PMC9765702; doi:10.1128/mbio.02688-22)
Supplement: TABLE S3 [file mbio.02688-22-s0008.docx]

Table S3. **Predicted functional proteins unique to the clinical isolates**.

| **Category** | **Mbale** | **Mbale2** | **Mbale3** |
| --- | --- | --- | --- |
| **Secondary Metabolism** | Phosphoribosylanthranilate isomerase (EC 5.3.1.24) | Phosphoribosylanthranilate isomerase (EC 5.3.1.24) | Phosphoribosylanthranilate isomerase (EC 5.3.1.24) |
|  | Streptolysin S export transmembrane permease (SagH) | Streptolysin S export transmembrane permease (SagH) |  |
|  | Lantibiotic biosynthesis dihydropyridine synthase, TsrD family |  |  |
| **Phosphorus Metabolism** | 2-aminoethylphosphonate:pyruvate aminotransferase (EC 2.6.1.37) | 2-aminoethylphosphonate:pyruvate aminotransferase (EC 2.6.1.37) | 2-aminoethylphosphonate:pyruvate aminotransferase (EC 2.6.1.37) |
|  | Phosphoenolpyruvate phosphomutase (EC 5.4.2.9) | Phosphoenolpyruvate phosphomutase (EC 5.4.2.9) | Phosphoenolpyruvate phosphomutase (EC 5.4.2.9) |
|  | Phosphonopyruvate decarboxylase (EC 4.1.1.82) | Phosphonopyruvate decarboxylase (EC 4.1.1.82) | Phosphonopyruvate decarboxylase (EC 4.1.1.82) |
| **Clustering-based subsystems** | TldD protein, part of TldE/TldD proteolytic complex | FIG007959: peptidase, M16 family | FIG007959: peptidase, M16 family |
|  |  |  | TldD protein, part of TldE/TldD proteolytic complex |
|  |  |  | Magnesium and cobalt efflux protein CorC |
|  |  |  | Cell division protein FtsH (EC 3.4.24.-) |
| **Carbohydrates** | Citrate lyase alpha chain (EC 4.1.3.6) |  | Citrate lyase alpha chain (EC 4.1.3.6) |
|  | Citrate lyase beta chain (EC 4.1.3.6) | Beta-glucosidase (EC 3.2.1.21) | Citrate lyase beta chain (EC 4.1.3.6) |
|  | [Citrate [pro-3S]-lyase] ligase (EC 6.2.1.22) | Mannose-1-phosphate guanylyltransferase (EC 2.7.7.13 ) | [Citrate [pro-3S]-lyase] ligase (EC 6.2.1.22) |
|  | NAD-dependent malic enzyme (EC 1.1.1.38) |  | NAD-dependent malic enzyme (EC 1.1.1.38) |
|  |  |  | PTS system, N-acetylglucosamine-specific IIB component (EC 2.7.1.69) |
|  |  |  | Glycolate dehydrogenase (EC 1.1.99.14), subunit GlcD |
|  |  |  | D-amino acid dehydrogenase small subunit (EC 1.4.99.1) |
|  |  |  | 3-hydroxybutyryl-CoA epimerase (EC 5.1.2.3) |
|  |  |  | D-lactate dehydrogenase (EC 1.1.1.28) |
|  |  |  | Propionate catabolism operon regulatory protein PrpR |
|  |  | Phosphoglycerate mutase (EC 5.4.2.1) | Phosphoglycerate mutase (EC 5.4.2.1) |
| ***Phage derived*** | *Phage major capsid protein** | Phage major capsid protein | Phage portal protein |
|  | *Phage major capsid protein** |  | Prophage Clp protease-like protein |
|  | *Prophage Clp protease-like protein* |  |  |
|  | Phage minor capsid protein |  |  |
|  | Phage terminase, small subunit |  |  |
| **Membrane Transport and Secretion systems** | Recombinational DNA repair protein RecT (prophage associated) | Leader peptidase (Prepilin peptidase) (EC 3.4.23.43) | Leader peptidase (Prepilin peptidase) (EC 3.4.23.43) |
|  | Leader peptidase (Prepilin peptidase) (EC 3.4.23.43) | Type IV fimbrial assembly protein PilC | N-methyltransferase (EC 2.1.1.-) |
|  | Type IV fimbrial assembly protein PilC* | Twitching motility protein PilT | Twitching motility protein PilT |
|  | Twitching motility protein PilT | N-methyltransferase (EC 2.1.1.-) | Type IV fimbrial assembly protein PilC |
| **DNA Metabolism** |  | ATP-dependent DNA ligase (EC 6.5.1.1) |  |
|  | ATP-dependent DNA ligase (EC 6.5.1.1) | DNA-cytosine methyltransferase (EC 2.1.1.37) |  |
| **Cell Wall and Capsule** | Putative deoxyribonuclease similar to YcfH, type 4 | Teichoic acid translocation permease protein TagG | Teichoic acid translocation permease protein TagG |
|  | Teichoic acid translocation permease protein TagG | dTDP-Rha:A-D-GlcNAc-diphosphoryl polyprenol, A-3-L-rhamnosyl transferase WbbL |  |
| **Amino Acids and Derivatives** |  | Isochorismate synthase (EC 5.4.4.2) | Isochorismate synthase (EC 5.4.4.2) |
|  |  |  | Spermidine Putrescine ABC transporter permease component PotB (TC 3.A.1.11.1) |
| **Cofactors, Vitamins, Prosthetic Groups, Pigments** |  | 4-hydroxythreonine-4-phosphate dehydrogenase (EC 1.1.1.262) | 4-hydroxythreonine-4-phosphate dehydrogenase (EC 1.1.1.262) |
|  |  | Predicted transcriptional regulator of pyridoxine metabolism | Predicted transcriptional regulator of pyridoxine metabolism |
| **Metabolism of Aromatic Compounds** |  | Beta-ketoadipate enol-lactone hydrolase (EC 3.1.1.24) | 4-hydroxyphenylacetate 3-monooxygenase (EC 1.14.13.3) |
| **Miscellaneous** |  | L-alanine-DL-glutamate epimerase |  |
| **Protein Metabolism** |  | TsaB protein, required for threonylcarbamoyladenosine (t(6)A) formation in tRNA | Ribosomal subunit interface protein |
| **RNA Metabolism** |  | Ribonuclease J2 (endoribonuclease in RNA processing) | Ribonuclease J2 (endoribonuclease in RNA processing) |
|  |  | LSU m3Psi1915 methyltransferase RlmH |  |
| **Stress Response** |  | Outer membrane stress sensor protease DegS | Outer membrane stress sensor protease DegS |
|  |  |  | Choline-sulfatase (EC 3.1.6.6) |
| **Virulence, Disease and Defense** |  | Multidrug efflux transporter MdtP | Multidrug efflux transporter MdtP |
| **Sulfur Metabolism** |  | Beta-galactosidase (EC 3.2.1.23) | Beta-galactosidase (EC 3.2.1.23) |
| **Nucleosides and Nucleotides** |  |  | Xanthine and CO dehydrogenases maturation factor, XdhC/CoxF family |
|  |  |  | Xanthine dehydrogenase iron-sulfur subunit (EC 1.17.1.4) |
|  |  |  | Xanthine dehydrogenase, FAD binding subunit (EC 1.17.1.4) |
|  |  |  | Xanthine dehydrogenase, molybdenum binding subunit (EC 1.17.1.4) |
| **Regulation and Cell signaling** |  |  | Phosphotransferase system, phosphocarrier protein HPr |
| **Respiration** |  |  | Fumarate reductase flavoprotein subunit (EC 1.3.99.1) |
|  |  |  |  |
