# Supplementary material for: Type IV Pili Are a Critical Virulence Factor in Clinical Isolates of Paenibacillus thiaminolyticus
Source: mBio. 2022 Nov 14;13(6):e02688-22. doi: 10.1128/mbio.02688-22 (PMC9765702; doi:10.1128/mbio.02688-22)
Supplement: FIG S2 [file mbio.02688-22-s0002.docx]

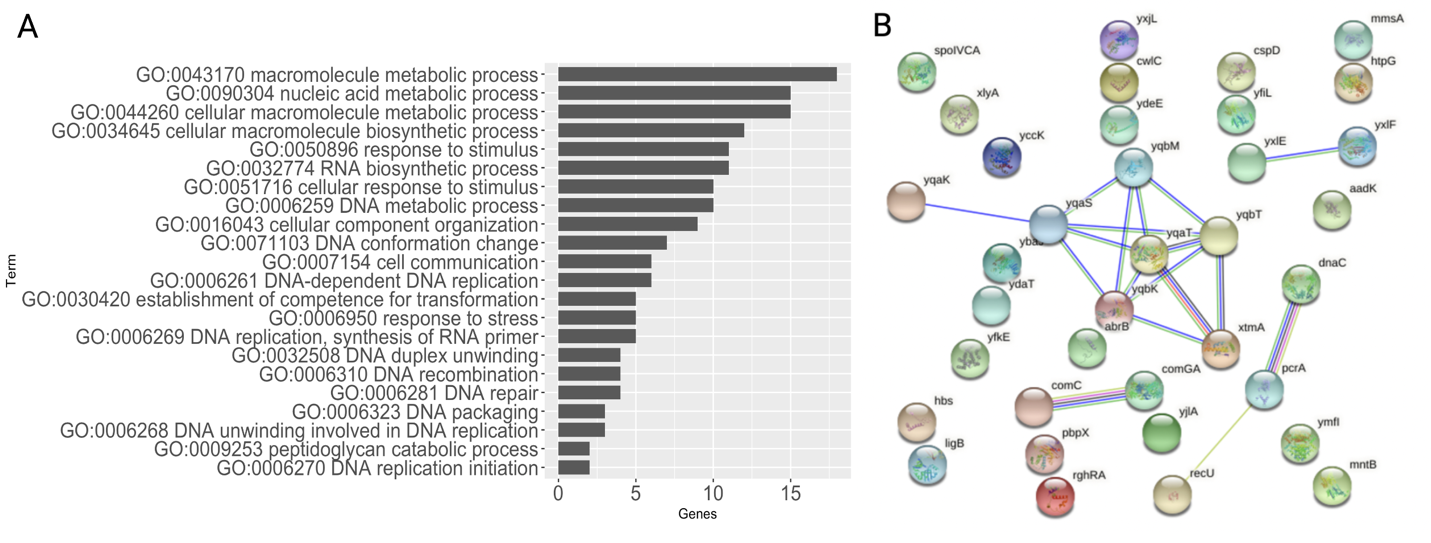


Fig S2. **Gene ontology terms of annotated insertions from MGEFinder**. A) Bar plot of the numbers of genes identified to each gene ontology terms. B) STRING analysis of the 342 unique clinical isolate proteins identified a network of protein interactions between uncharacterized proteins (YqaK, YgaS, YbcM, YqaT, YqbK,YqbT) and phage terminase (XtmA), along with additional protein-protein interactions related to competence (ComC and ComA), DNA repair (RecA, PcrA, and DnaC) and protein transport (YxlE and YxlF).
