# Supplementary material for: Type IV Pili Are a Critical Virulence Factor in Clinical Isolates of Paenibacillus thiaminolyticus
Source: mBio. 2022 Nov 14;13(6):e02688-22. doi: 10.1128/mbio.02688-22 (PMC9765702; doi:10.1128/mbio.02688-22)
Supplement: TABLE S4 [file mbio.02688-22-s0009.docx]

Table S4. **Antibiotic susceptibility testing results of the three clinical isolates and the reference strain**.

|  | Mbale2 | | Mbale3 | | Mbale | | B-4156 | |
| --- | --- | --- | --- | --- | --- | --- | --- | --- |
|  | MIC (µg/mL) | Interpretation* | MIC (µg/mL) | Interpretation | MIC (µg/mL) | Interpretation | MIC (µg/mL) | Interpretation |
| Ampicillin | 1 | R | 0.5 | R | 0.12 | S | 0.032 | S |
| Ceftriaxone | 0.25 | ** | 0.06 | ** | 0.03 | ** | 0.032 | ** |
| Ciprofloxacin | 0.12 | S | 0.12 | S | 0.12 | S | 0.125 | S |
| Clindamycin | 1 | I | 4 | R | 1 | I | 0.5 | S |
| Gentamicin | 2 | S | 2 | S | 2 | S | 2 | S |
| Penicillin | 0.5 | R | 0.25 | R | 0.12 | S | 0.032 | S |
| Meropenem | 0.5 | S | 0.5 | S | 0.25 | S | 0.5 | S |
| Tetracycline | 2 | S | 2 | S | 0.5 | S | 4 | S |
| Vancomycin | 8 | Nonsusceptible | 8 | Nonsusceptible | 8 | Nonsusceptible | 8 | Nonsusceptible |

* R, resistant, S, sensitive, or **No interpretation by CLSI guidelines
