# Supplementary material for: Type IV Pili Are a Critical Virulence Factor in Clinical Isolates of Paenibacillus thiaminolyticus
Source: mBio. 2022 Nov 14;13(6):e02688-22. doi: 10.1128/mbio.02688-22 (PMC9765702; doi:10.1128/mbio.02688-22)
Supplement: FIG S3 [file mbio.02688-22-s0003.docx]

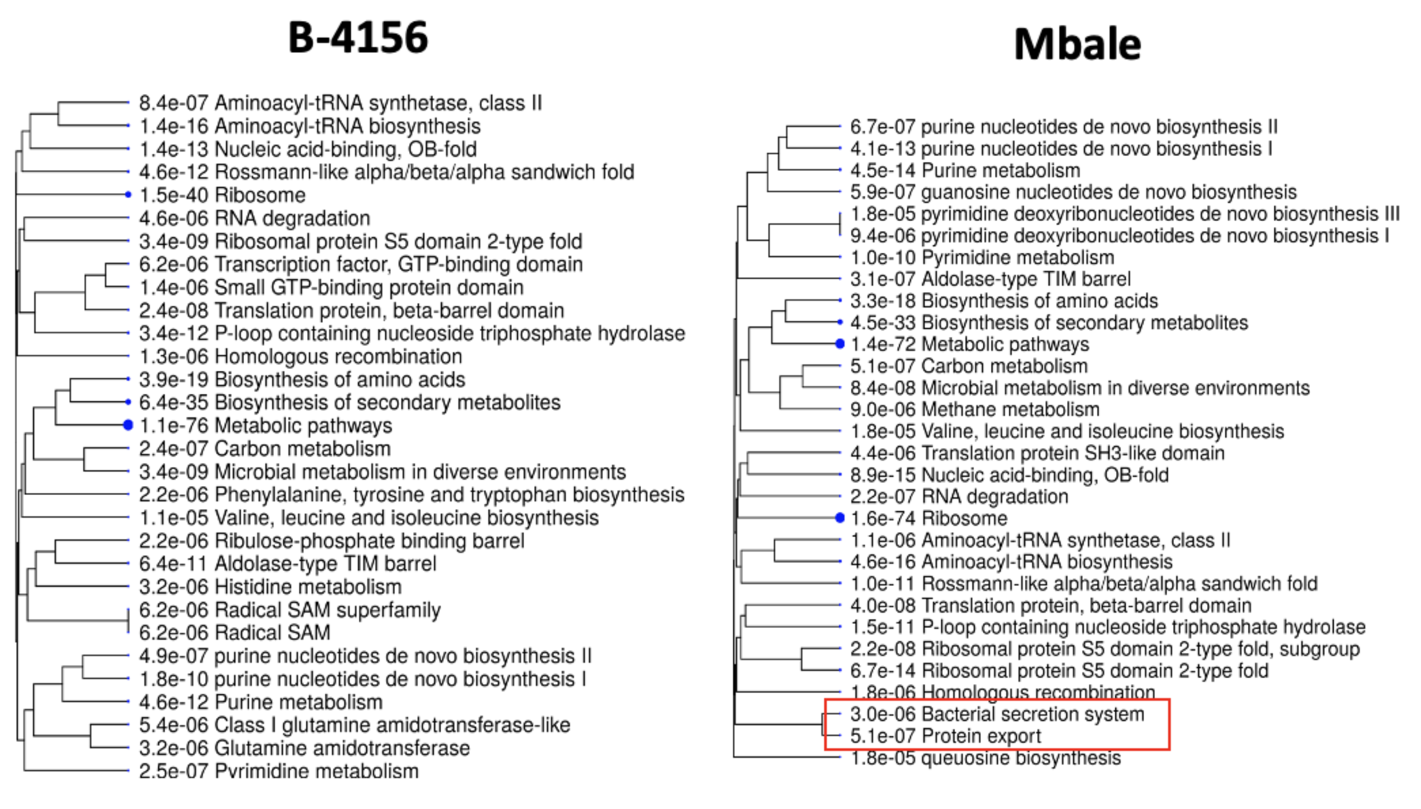


Fig S3**. Gene ontology of the unique proteins in each isolate.** Gene ontology of the 450 proteins uniquely identified in proteomic data for the B-4156 strain (left) and the 468 proteins unique to the Mbale strain (right). P-values indicate the extent of enrichment of the genes of each indicated ontological group in the set of genes unique to each of the strains. Bacterial secretion system and protein export genes related to the SecA pathway uniquely represented in the Mbale strain are highlighted in red.
