# Supplementary material for: Type IV Pili Are a Critical Virulence Factor in Clinical Isolates of Paenibacillus thiaminolyticus
Source: mBio. 2022 Nov 14;13(6):e02688-22. doi: 10.1128/mbio.02688-22 (PMC9765702; doi:10.1128/mbio.02688-22)
Supplement: FIG S4 [file mbio.02688-22-s0004.docx]

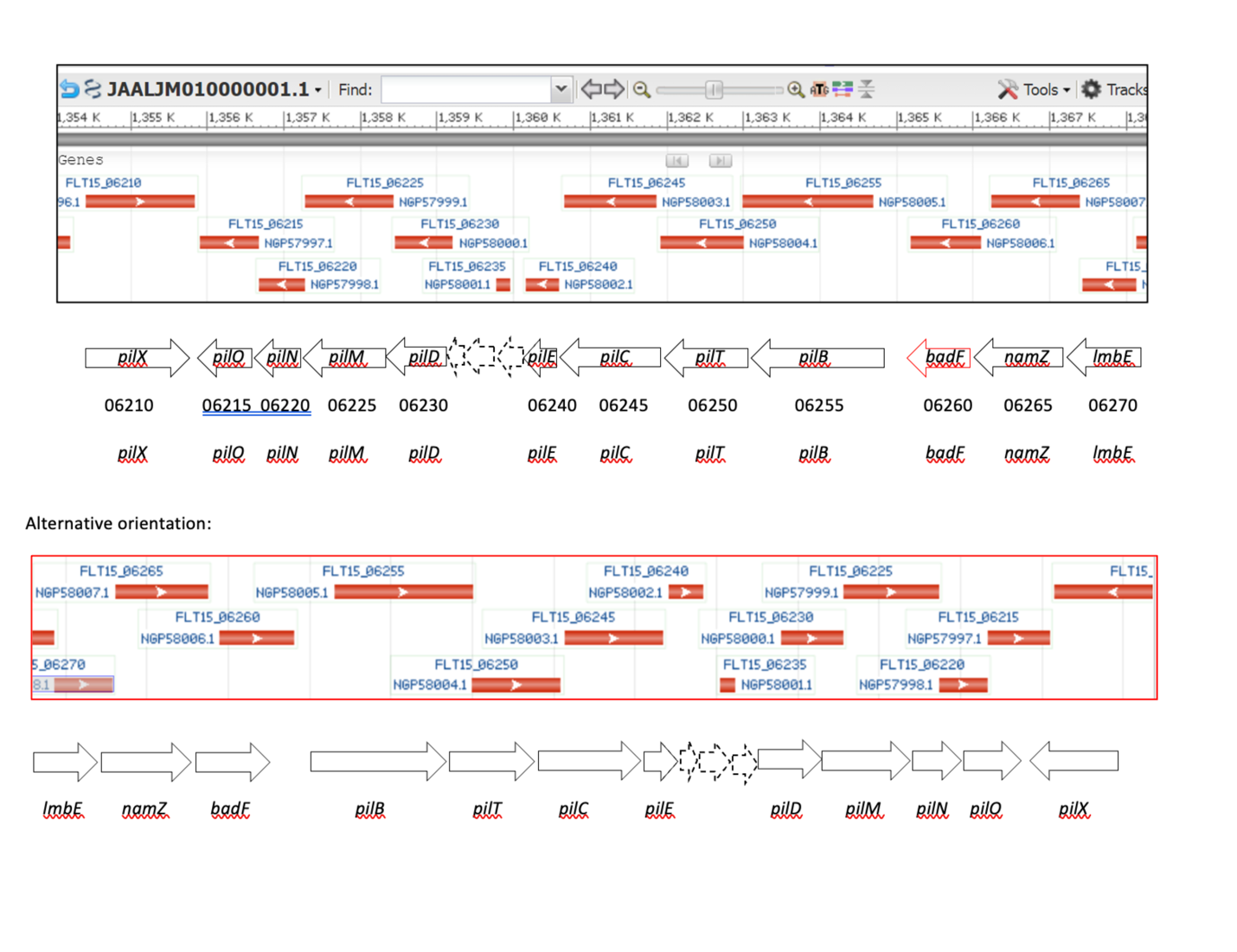


Fig S4. **Comparison between previously identified T4P operon and Mbale clinical isolate.** Comparison of the gene order in the *pil* operon of *P. thiaminolyticus* Mbale with the previously characterized *pil* operons from other Gram-positive bacteria (1, 2) **showed the closest match to the *pil* operons of *Clostridium cellulolyticum* H10 and *Bacillus* sp. NRRL B-14911. The latter strain was originally isolated from the Gulf of Mexico (3) but was subsequently reclassified as *Bacillus infantis* (4)**; its genome (GenBank accession number [CP006643.1](https://www.ncbi.nlm.nih.gov/nuccore/CP006643.1)) is currently the only complete genome sequence from that species. Remarkably, *B. infantis* was so named after being isolated from a case of neonatal sepsis in Busan, South Korea (5)**. Although not recognized at the time, T4P could have contributed to virulence in that case as well.**

1. Gurung I, Spielman I, Davies MR, Lala R, Gaustad P, Biais N, Pelicic V. 2016. Functional analysis of an unusual type IV pilus in the Gram-positive Streptococcus sanguinis. Mol Microbiol 99:380-92.

2. Imam S, Chen Z, Roos DS, Pohlschroder M. 2011. Identification of surprisingly diverse type IV pili, across a broad range of gram-positive bacteria. PLoS One 6:e28919.

3. Siefert JL, Larios-Sanz M, Nakamura LK, Slepecky RA, Paul JH, Moore ER, Fox GE, Jurtshuk P, Jr. 2000. Phylogeny of marine Bacillus isolates from the Gulf of Mexico. Curr Microbiol 41:84-8.

4. Massilamany C, Mohammed A, Loy JD, Purvis T, Krishnan B, Basavalingappa RH, Kelley CM, Guda C, Barletta RG, Moriyama EN, Smith TP, Reddy J. 2016. Whole genomic sequence analysis of Bacillus infantis: defining the genetic blueprint of strain NRRL B-14911, an emerging cardiopathogenic microbe. BMC Genomics 17 Suppl 7:511.

5. Ko KS, Oh WS, Lee MY, Lee JH, Lee H, Peck KR, Lee NY, Song JH. 2006. Bacillus infantis sp. nov. and Bacillus idriensis sp. nov., isolated from a patient with neonatal sepsis. Int J Syst Evol Microbiol 56:2541-2544.
