# Supplementary material for: Type IV Pili Are a Critical Virulence Factor in Clinical Isolates of Paenibacillus thiaminolyticus
Source: mBio. 2022 Nov 14;13(6):e02688-22. doi: 10.1128/mbio.02688-22 (PMC9765702; doi:10.1128/mbio.02688-22)
Supplement: FIG S5 [file mbio.02688-22-s0005.docx]

Fig S5. **Validation and schema of T4P knockout in Mbale isolate.** A) PCR products were run on a 1% agarose gel spanning the pilC, pilT, and pilB genes. Comparing the sizes of the PCR products of the wildtype (WT) compared to the knockout (KO) identifies a 4500 bp knockout. B) Schematic including the guide RNA (sgRNA, red) for the CRISPR-Cas9 cut in the genome and primers with appropriate locations in the genome that were used for the PCR confirmation.
